# The cellular prion protein interacts with and promotes the activity of Na,K-ATPases

**DOI:** 10.1371/journal.pone.0258682

**Published:** 2021-11-30

**Authors:** Declan Williams, Mohadeseh Mehrabian, Hamza Arshad, Shehab Eid, Christopher Sackmann, Wenda Zhao, Xinzhu Wang, Farinaz Ghodrati, Claire E. Verkuyl, Joel C. Watts, Gerold Schmitt-Ulms

**Affiliations:** 1 Tanz Centre for Research in Neurodegenerative Diseases, University of Toronto, Toronto, Ontario, Canada; 2 Department of Laboratory Medicine & Pathobiology, University of Toronto, Toronto, Ontario, Canada; 3 Department of Biochemistry, University of Toronto, Toronto, Ontario, Canada; Ruhr University Bochum, GERMANY

## Abstract

The prion protein (PrP) is best known for its ability to cause fatal neurodegenerative diseases in humans and animals. Here, we revisited its molecular environment in the brain using a well-developed affinity-capture mass spectrometry workflow that offers robust relative quantitation. The analysis confirmed many previously reported interactions. It also pointed toward a profound enrichment of Na,K-ATPases (NKAs) in proximity to cellular PrP (PrP^C^). Follow-on work validated the interaction, demonstrated partial co-localization of the ATP1A1 and PrP^C^, and revealed that cells exposed to cardiac glycoside (CG) inhibitors of NKAs exhibit correlated changes to the steady-state levels of both proteins. Moreover, the presence of PrP^C^ was observed to promote the ion uptake activity of NKAs in a human co-culture paradigm of differentiated neurons and glia cells, and in mouse neuroblastoma cells. Consistent with this finding, changes in the expression of 5’-nucleotidase that manifest in wild-type cells in response to CG exposure can also be observed in untreated PrP^C^-deficient cells. Finally, the endoproteolytic cleavage of the glial fibrillary acidic protein, a hallmark of late-stage prion disease, can also be induced by CGs, raising the prospect that a loss of NKA activity may contribute to the pathobiology of prion diseases.

## Introduction

Prion diseases are incurable neurodegenerative diseases in humans and animals, including sheep, cattle and cervids. The etiology of these devastating diseases centers on the prion protein, whose normal cellular form (PrP^C^) is expressed in most vertebrate cells. In the disease, PrP^C^ undergoes a conformational change to PrP^Sc^ that endows the protein with distinct physicochemical properties. PrP^Sc^ can seed the conversion of other PrP^C^ molecules through templated conversion, thereby causing the accumulation of PrP^Sc^ and the spread of the disease [1]. Although progress has been made [2–4], the precise mechanisms through which PrP^Sc^ causes toxicity to brain cells are still being investigated. Because PrP-deficient cells and mice exhibit no overt phenotypes [5], it is widely assumed that the toxicity of PrP^Sc^ involves a gain-of-toxic function [6]. However, it is also apparent that the expression of PrP^C^ is critical for cellular toxicity to manifest in prion diseases [7]. Consequently, ongoing research aims to decipher the molecular mechanism by which PrP^C^ contributes to the disease with the focus on blocking the toxicity of PrP^Sc^.

It is conceivable that the molecular interactions of PrP with other nearby factors play a role in each of these processes. Therefore, considerable efforts have been invested in both the characterization of the membrane microdomain in which PrP^C^ resides and the identification of its interactions. Although no shortage of PrP^C^ binders have been described [8], this body of literature is somewhat fragmented and inconsistent. To shed light on whether differences in methodology or experimental paradigms are to blame, we recently undertook the first side-by-side analysis of proteins that were *in vivo* crosslinked to PrP^C^ in four distinct mouse cell models using identical workflows [9]. This work corroborated the notion that the molecular environment of PrP^C^ can vary from cell model to cell model but also led to two main conclusions. First, it placed emphasis on the previously reported interaction of PrP^C^ with the neural cell adhesion molecule 1 (Ncam1) [10–12] by revealing it to be the only protein PrP^C^ could readily be crosslinked to in all four models tested. Second, it uncovered a shared functional specialization of the molecular environment of PrP^C^ in all four models, namely its close proximity to cellular factors known to modulate the activity of Tgfb1 and integrin sister signaling complexes. Importantly however, the paradigm-specific differences in the precise composition of PrP^C^ interactors provided a strong argument for revisiting the molecular environment of PrP^C^ in the brain, the organ central to the pathobiology of prion diseases.

**Fig 1:**
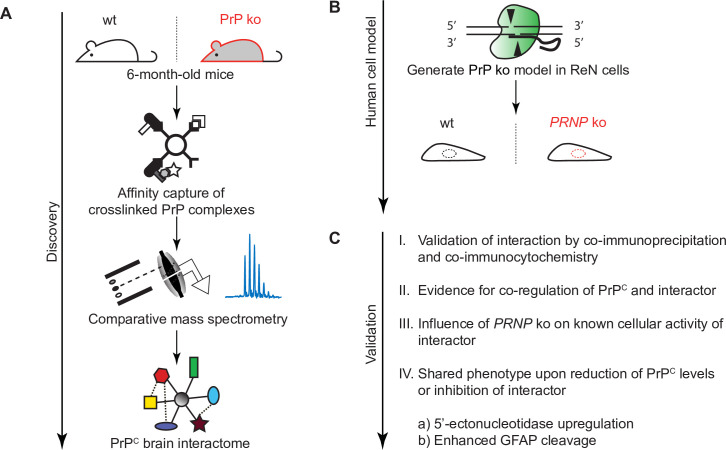
Study overview. (A) Workflow of brain PrP interactome discovery analysis. (B) CRISPR-Cas9-based knockout of PrP in multi-potent human neural stem cell line. (C) Validation of top-listed PrP interactor.

Here, we undertook such an analysis using leading-edge technology and workflows (**Fig 1**). The results pointed toward a stronger than previously appreciated relative abundance of Na,K-ATPases (NKAs) in close proximity to PrP^C^. Not only were NKAs observed to be the most abundant brain proteins in proximity to PrP^C^ but we show that this close relationship translates into a correlated response of NKAs and PrP^C^ to cardiac glycosides (CGs), a well-characterized class of NKA inhibitor. Moreover, PrP deficiency reduced NKA-dependent ion uptake activity in a human co-culture model of neurons and astrocytes and in mouse neuroblastoma cells. Strikingly, both cellular exposure to non-toxic levels of CGs or PrP deficiency caused an increase in the levels of a 60 kDa protein band, which we identified to be composed of the GPI-anchored 5’-nucleotidase, also known as CD73. Finally, the sustained exposure of differentiated human neurons and astrocytes to non-toxic levels of CGs caused an increase in the levels of glial fibrillary acidic protein (GFAP) and its calpain cleavage products, reminiscent of similar molecular signatures in PrP^Sc^-infected brains.

## Results

### The molecular environment of PrP^C^ in mouse brains is dominated by NKAs

**Fig 2:**
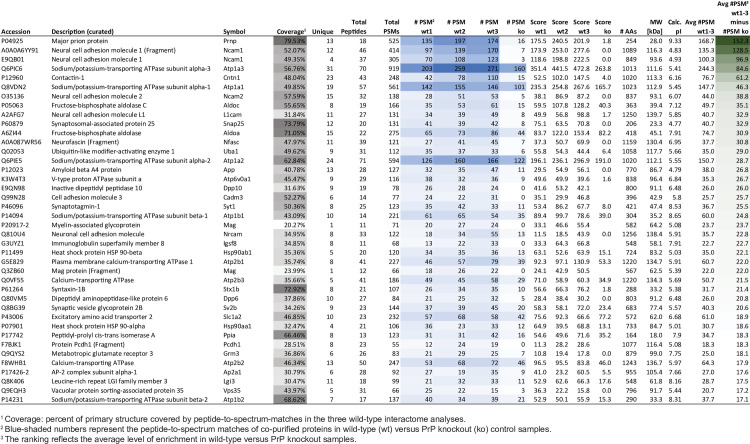
PrP interactors in mouse brain. Truncated list of proteins observed in eluate fractions in this study. The intensity of (i) gray shading reflects the sequence coverage, (ii) blue shading reflects the number of peptide-to-spectrum matches detected, and (iii) green shading reflects the relative enrichment in wild-type versus PrP knockout samples.

To facilitate the capture of proteins that reside *in vivo* in the mouse brain in spatial proximity to PrP^C^, we stabilized protein interactions prior to brain homogenization through time-controlled transcardiac perfusion crosslinking [13, 14]. To maximize interactome coverage, equal aliquots of trypsin digested eluate samples were initially subjected to separate liquid chromatography/tandem mass spectrometry analyses and relative quantitations were based on the spectral counting method [15]. Three biological replicates constituted separate PrP^C^ co-immunoprecipitations from distinct wild-type brains, with identically processed samples from *Prnp*^*-/-*^ brains serving as negative controls. The spectral counting-based analysis of unlabeled tryptic digests of co-immunoprecipitates revealed 384 proteins whose levels were enriched in PrP-specific samples relative to *Prnp*^-/-^ derived controls (**Fig 2**, **S1 Table**). Indicative of low non-specific protein contaminant levels, the prion protein was observed with an average of 168 peptide-to-spectrum matches (PSMs) in wild-type and only 16 PSMs in *Prnp*^-/-^ derived control eluates, giving rise to the highest difference count of 152 PSMs (wild-type minus control) observed for any protein in the dataset. When all proteins were sorted by their difference in spectral counts, the neural cell adhesion molecule 1 (Ncam1) was the most co-enriched interactor, and its paralog Ncam2, as well as the closely related proteins contactin-1, the neural cell adhesion molecule L1 and neurofascin, were amongst the top ten-listed proteins. Amongst the top ten group of PrP candidate interactors were also two alpha subunits of NKAs, namely alpha-1 and alpha-3, the latter known to be predominantly expressed in neurons. Additional NKA subunits, comprising Atp1a2, Atp1b1 and Atp1b2 were among the 40 most co-enriched proteins. However, whereas Ncam1 and other members of the immunoglobulin superfamily, including cell adhesion molecule 3 (Cad3), myelin-associated glycoprotein (Mag), neuronal cell adhesion molecule (Nrcam) and immunoglobulin superfamily member 8 (Igsf8), exhibited low non-specific binding to the affinity matrix, NKA subunits were also relatively abundant in the negative control, consistent with the interpretation that the presence of these pumps in the PrP-specific eluates reflects both PrP-dependent and PrP-independent binding to the affinity matrix. Finally, the list of the 40 most co-enriched proteins also contained: 1) subunits of fructose-bisphosphate aldolase (Aldoa, Aldoc); 2) synaptic fusion complex subunits (Snap25, Syt1, Stx1b, Sv2b); 3) dipeptidyl peptidases (Dpp6 and Dpp10); 4) a ubiquitin-like modifier-activating enzyme 1 (Uba1); 5) the amyloid precursor protein (APP); 6) subunits of other ATPases (Atp6v0a1, Atp2b1, Atp2b2); 7) heat shock protein 90 (Hsp90ab1 and Hsp90aa1); 8) the excitatory amino acid transporter 2 (Slc1a2); 9) peptidyl-prolyl cis-trans isomerase A; and 10) the metabotropic glutamate receptor.

**Fig 3:**
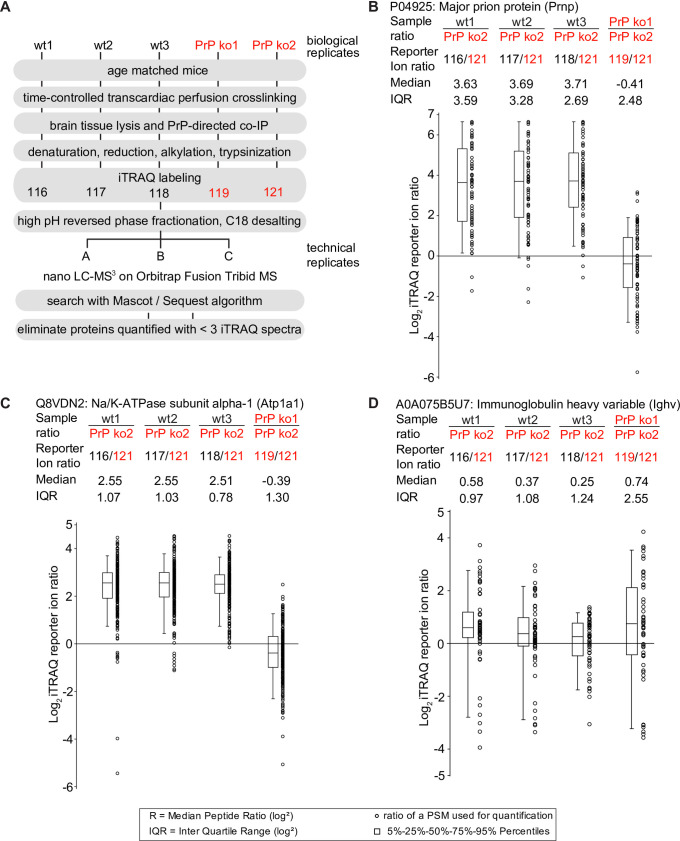
Evidence for selective PrP co-enrichment of NKAs in mouse brain. (A) Quantitative mass spectrometry sample processing workflow. (B-D) Side-by-side box plots depicting relative peptide enrichment levels in tryptic digests of PrP brain interactome eluates. (B) Selective enrichment of PrP-derived peptides in wild-type eluates (not in PrP ko eluates). (C) Exemplary PrP co-enrichment of the NKA alpha-1 subunit ATP1A1 (note that other NKA subunits were also observed to co-enrich with PrP; see **Fig 2** for details). (D) Immunoglobulin levels were observed at similar abundance levels in all samples, including interactome eluates from PrP ko cells, consistent with the interpretation that levels of the bait antibody were balanced in all samples.

Next, isobaric labeling with iTRAQ reagents followed by multiplexing was undertaken with a view to minimize run-to-run variance and consolidate relative quantifications of the more abundant proteins [16] (**Fig 3A**). Identical procedures had previously been successfully employed to study PrP^C^ interactors in four murine cell models [9]. The relative enrichment of proteins in iTRAQ-labeled wild-type samples versus PrP knockout controls was computed using the ‘Reporter Ion Quantifier’ algorithm within Proteome Discoverer. The ‘Major prion protein’ was again amongst the proteins identified with the most PSMs (>400 PSMs), a correlate for relative abundance [15]. More than seventy iTRAQ-reporter ion ratios, collected after the high-energy fragmentation of the most intense ions observed in a given PSM, validated the expected PrP enrichment in wild-type versus *Prnp*^-/-^ derived samples (**Fig 3B**). Note that despite the absence of PrP in the *Prnp*^-/-^ derived samples, the average PrP enrichment ratio is not infinite (as would be mathematically expected) but approximates a value of 12 (Log_2_ 3.6) due to the existence of background noise in all iTRAQ reporter ion channels, purity limitations of iTRAQ chemistry and the inadvertent co-isolation of contaminating ions observed in complex peptide mixtures that work together to artificially limit the dynamic range (**S1 Fig, panels A and B**). Ncam1 was also again highly enriched, its peptides exhibiting a median >8-fold (Log_2_ 3.0) enrichment over negative controls (**S1C Fig**). Whereas the iTRAQ results for α-subunits of NKAs validated their co-enrichment with PrP (e.g., Log_2_ 2.5 for Atp1a1) (**Fig 3C**), peptides belonging to the D18 antibody that was used for the co-affinity capture were observed at similar levels in all samples (**Fig 3D**). Taken together, these data corroborated Ncam1 as a predominant PrP interactor but also put a spotlight on NKAs, indicating that these pumps may be abundant in spatial proximity to PrP^C^ in the brain.

### NKAs and PrP interact and their levels are somewhat correlated

**Fig 4:**
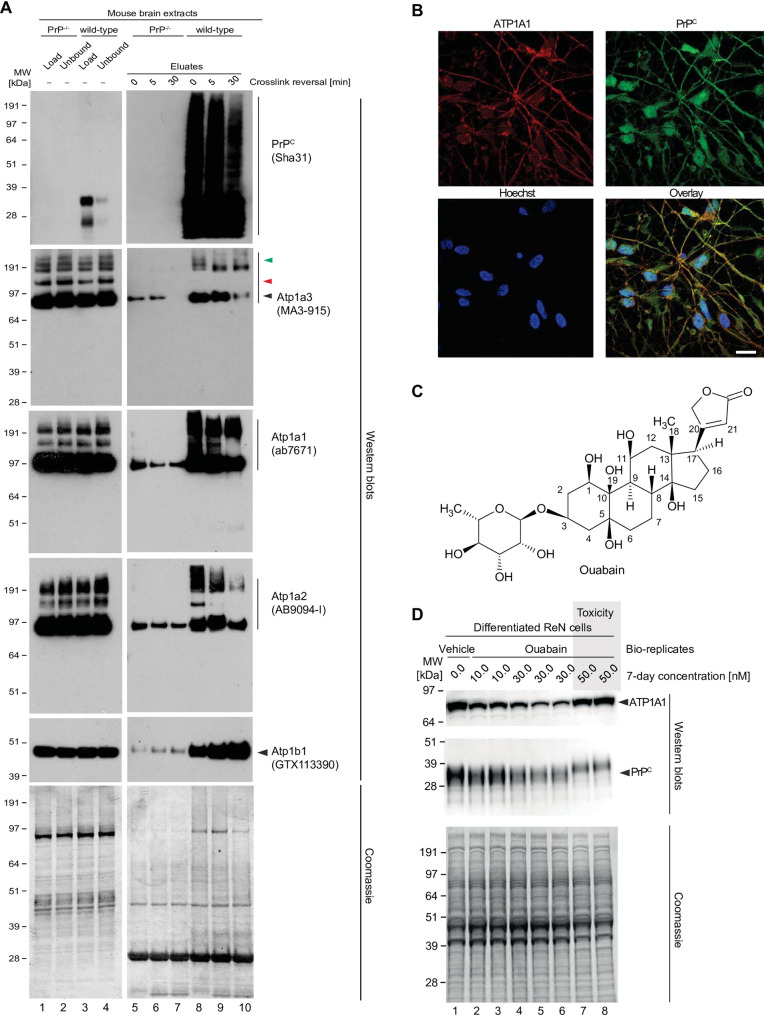
Validation of NKA binding to PrP. (A) Western blot-based validation of co-immunoprecipitation of NKA subunits with PrP. (B) Evidence of partial cellular co-localization of PrP^C^ and ATP1A1 by co-immunocytochemical analysis of ReN VM cells, with Hoechst stain serving as the nuclear counter stain. Scale bar: 10 nm. (C) Chemical structure of the NKA inhibitor ouabain. (D) Parallel ouabain concentration-dependent effects on NKA and PrP^C^ levels. Exposure of differentiated ReN VM cells to ouabain at concentrations up to 50 nM causes a biphasic effect on PrP^C^ and ATP1A1 levels, with concentrations up to 30 nM leading to a ouabain concentration-dependent reduction in steady-state levels of both proteins, and exposure to 50 nM provoking a reproducible slowing of PrP^C^ during SDS-PAGE separation that parallels an increase in steady-state ATP1A1 levels.

To validate the putative interaction between PrP^C^ and NKAs, brain homogenates generated from *in vivo* crosslinked mouse brains were subjected to small-scale PrP-directed co-immunoprecipitation followed by western blot analyses. Samples produced side-by-side from *Prnp*^-/-^ mice served as negative controls (**Fig 4A**). This experiment corroborated the observation that small amounts of alpha and beta subunits of NKAs bind non-specifically to the affinity capture matrix, as the mass spectrometry analyses had revealed. Critically, the western blot data also validated the PrP-dependent binding of NKAs. Moreover, a comparison of the banding pattern of extract and eluate fractions obtained with the Atp1a3-directed western blot provided evidence that the presence of NKAs in PrP-specific eluate fractions cannot be ascribed to trivial carryover of NKAs from extracts into eluates. If the latter had been the case, input and eluate signals would have been expected to give rise to the same NKA alpha subunit western blot band patterns, which was not observed. Moreover, heating the eluate fractions at 90°C in the presence of β-mercaptoethanol for 5 or 30 minutes caused a subset of high molecular weight bands to disappear, consistent with the interpretation that they comprised crosslinked products.

Next, we investigated if PrP^C^ co-localizes with NKAs in human cells. To this end, we employed a neuronal cell model (Ren VM cells) known to harbor a stable human male karyotype (46, XY) and to differentiate into co-cultures of neurons and glia cells within one week of growth factor removal [17], an observation we recently validated in our own works [18]. In these analyses we focused on ATP1A1 because it is the only NKA alpha-subunit known to be expressed in both neurons and glia cells. As expected, these co-immunocytochemical analyses indicated that both ATP1A1 and PrP^C^ were localized to both neurites and cell bodies. The overlay analysis indicated a Pearson correlation coefficient of 0.64 for signals derived from the detection of these two proteins in this cell model (**Fig 4B,** see also **S2 Fig, panels A-C**).

The expression of proteins that engage in functional associations is often coordinated. To assess the extent to which the biology of PrP^C^ and NKAs may be interlinked, we set out to explore the effect of cardiac glycosides (CGs) on the steady-state levels of NKAs, their natural targets, and on PrP^C^. The experiment made use of ouabain, a plant-derived CG (**Fig 4C**), because it is understood to have low membrane penetrance and therefore may predominantly affect NKAs at the cell surface. Perhaps not surprisingly, one-week exposure of differentiated ReN VM cells to a range of nanomolar concentrations of ouabain reproducibly altered levels of NKAs, here shown for the ATP1A1 subunit. More specifically, we observed a bimodal effect, whereby exposure of cells to low nanomolar levels of ouabain (approximately up to its IC50 of 15–41 nM [19]) resulted in a ouabain concentration-dependent depletion of ATP1A1, yet in the presence of mildly toxic 50 nM ouabain concentrations, ATP1A1 levels bounced back. Interestingly, levels of PrP^C^ correlated closely with ATP1A1 levels in the presence of non-toxic ouabain concentrations. No such correlation was observed for a majority of other proteins in the cell extract, as evidenced by Coomassie analyses. Intriguingly, at toxic ouabain concentrations, PrP^C^ was observed to migrate slower during denaturing SDS-PAGE separation, reminiscent of its migration under conditions that increase its abundance of complex N-glycans (**Fig 4D**). The enzymatic removal of

N-glycans revealed that PrP^C^ is present in ReN cells and that the apparent increase in molecular weight upon treatment with high concentrations of ouabain most likely reflects alterations to PrP^C^ glycosylation (**S2D Fig**). Interestingly, when comparing steady-state PrP^C^ levels in cells that were treated with 30 nM versus 50 nM Ouabain, a rebound in PrP^C^ signal intensity can often be observed at the higher cardiac glycoside concentration (**S2D Fig**).

To assess whether a transcriptional upregulation contributes to the increase in ATP1A1 and PrP^C^ levels observed in cells exposed to 50 nM Ouabain, we next undertook quantitative RT-qPCR analyses. mRNA levels of both proteins stayed unchanged during the course of a 7-day Ouabain treatment so long as the Ouabain concentration remained at or below 30 nM. In cells exposed to 50 nM Ouabain, transcript levels of both ATP1A1 and PRNP were significantly increased relative to vehicle-treated controls (**S2 Fig, panels E and F**). The mRNA levels of PPIA and GAPDH housekeeping genes, which were used to normalize results, remained unchanged at all conditions tested, suggesting that transcriptional upregulation of the ATP1A1 and PRNP genes contributes to the stronger ATP1A1 and PrP signal intensity observed at the 50 nM Ouabain concentration.

### PrP expression directly modulates NKA activity

**Fig 5:**
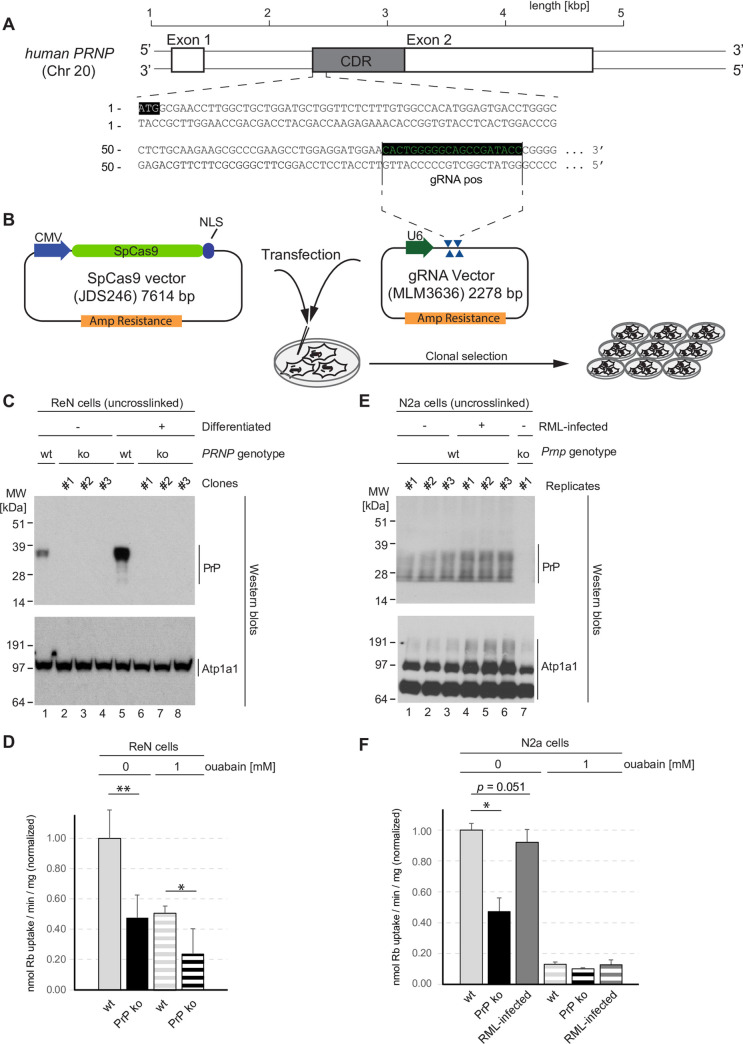
PrP deficiency or prion infection alter ^86^Rb^+^ uptake activity of NKA. (A) Design of gRNA targeting *PRNP* coding sequence. (B) CRISPR-Cas9-based knockout of PrP in ReN VM cells. (C) Validation of PrP knockout in ReN VM cells. Note that PrP deficiency has no effect on steady-state ATP1A1 levels in this model. (D) PrP knockout diminishes ^86^Rb^+^ uptake in ReN VM cells. Depicted are normalized mean plus standard deviation. (E) Increased steady-state ATP1A1 levels in RML-infected Neuro2a cells. (F) Similar to PrP knockout, RML-infection of Neuro2a cells compromises ^86^Rb^+^ uptake, albeit to a lesser extent. Statistical analyses in subpanels of this figure were based on the two-tailed t-test applied to three biological replicates.

To further investigate if the co-enrichment and partial co-localization of NKAs and PrP reflects a functional relationship between PrP^C^ and NKAs, we generated PrP-deficient derivative lines of the ReN VM cells. More specifically, we followed a CRISPR-Cas9 gene knockout strategy we had previously employed to generate several other PrP-deficient mouse cell lines [20] and targeted a genome stretch at the 5’ end of the PrP open reading frame (**Fig 5A**), capitalizing on the host-encoded non-homologous end-joining repair program to induce a frame shift and premature stop codon in the *PRNP* gene upon co-transfection of gRNAs and Cas9-expressing plasmids (**Fig 5B**). Immunoblot analyses validated the successful generation of several PrP knockout clones and established that the mere absence of PrP in this cell line did not alter the expression of the alpha-1 subunit of the NKA (ATP1A1) (**Fig 5C**). Next, we compared differentiated wild-type and PrP-deficient ReN VM cells in their NKA activity using a well-established radioisotope assay that capitalizes on the promiscuous uptake of rubidium (^86^Rb^+^)—as a stand-in for potassium—by the pump. To determine the contribution of NKAs to the total cellular ^86^Rb^+^ uptake activity, control samples were exposed to pump-saturating 1 mM concentrations of ouabain (**Fig 5D**). Wild-type ReN VM cells took up approximately 0.34 nmol of ^86^Rb^+^ per min and mg of total cellular protein. The complete block of NKAs reduced this uptake by 49%, indicating that uptake through NKAs accounts for approximately half of the total ^86^Rb^+^ internalization in this cell model.

Relative to wild-type cells, PrP-deficient cells exhibited a 52% reduction in the ^86^Rb^+^ uptake rate. Ouabain exposure of PrP ko cells further halved this uptake, bringing it to 24%, indicating that the presence of PrP somehow promotes ^86^Rb^+^ uptake. Moreover, these results suggested that a majority of the ^86^Rb^+^ uptake, which was promoted by the presence of PrP^C^, relied on NKAs. The latter point was even more convincingly conveyed when the analyses were repeated in N2a mouse neuroblastoma cells (**Fig 5, panels E and F**). In the latter model, the ouabain-resistant ^86^Rb^+^ uptake was observed to account for only 13% of total uptake, yet PrP-deficiency again reduced uptake rates by 53%, strongly arguing that PrP^C^ predominantly promoted the ouabain-sensitive uptake through NKAs (**Fig 5F**). An additional assessment of whether infection with mouse-adapted scrapie (RML) prions influences NKAs activity led to two observations. First, it suggested that levels of Atp1a1 may slightly increase in RML-infected N2a cells relative to non-infected cells, as evidenced by stronger signals in the western blot (**Fig 5E**). Rather than this slight increase in Atp1a1 levels leading to higher NKA activity, we observed an 8% reduction in total ouabain-sensitive ^86^Rb^+^ uptake rate in RML-infected N2a cells that narrowly missed the significance threshold (**Fig 5F**), consistent with the possibility that prion-infected cells may attempt to compensate for their loss in NKA activity by increasing the expression of these pumps. Results in this section pointed to a role of PrP^C^ promoting NKAs activity and suggested that either its removal (in PrP-deficient cells) or its perturbation (in prion disease) reduces the activity of NKAs, thereby mimicking the presence of CGs.

### Increased 5’-nucleotidase levels in the presence of CGs or in cells devoid of PrP

**Fig 6:**
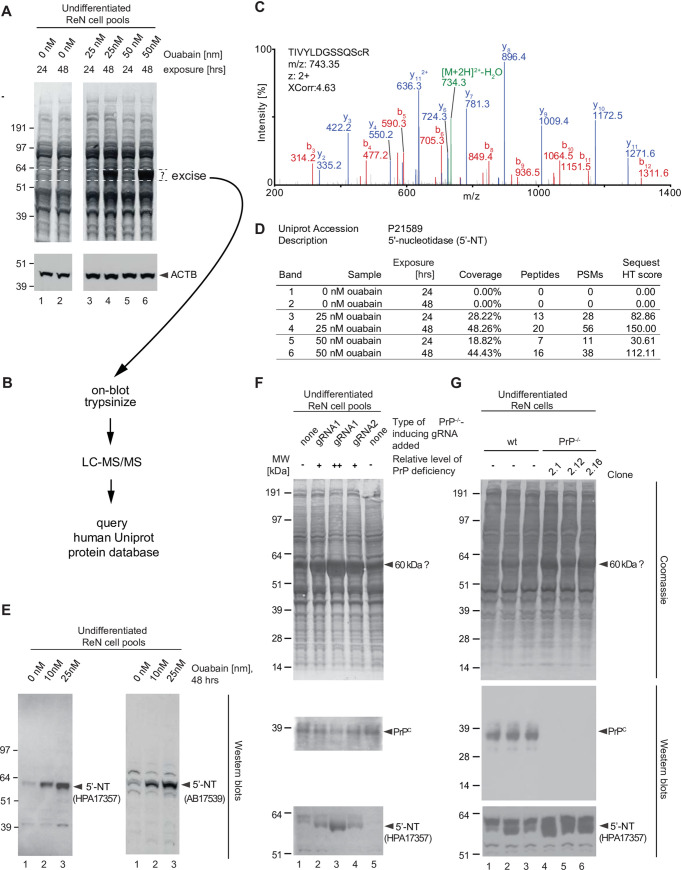
ReN VM cells respond to PrP-deficiency or cardiac glycoside exposure with an increase in the expression of a 60 kDa Coomassie-stained signal, originating from 5’-nucleotidase. (A) Observation of Coomassie-stained protein band signal at 60 kDa that is conspicuously increased in the presence of 48 hour exposure to ouabain. The blot depicting levels of actin B (ACTB) serves as an additional loading control of samples that had been adjusted for total protein levels. (B) Scheme for the on-blot digestion and mass spectrometry-based identification of the 60 kDa band-of-interest. (C) MS/MS spectrum documenting identification of 5-nucleotidase (5’-NT) as the protein underlying this band. (D) Spectral count comparison of MS-based 5’-NT identification from Western blot bands observed in the absence or presence of ouabain. (E) Western blot validation of changes to 5’-NT expression upon prolonged exposure to low levels of ouabain. (F) CRISPR/Cas9-mediated knockout of PrP mimics low levels of ouabain in its effect on steady-state 5’-NT levels. Initially, the changes in 5’-NT expression were studied in pools of CRISPR-Cas9 gene engineered ReN VM PrP^-/-^ cells generated with two distinct gRNAs. The pools differed in the percentages of cells depleted for PrP expression. Note that the 5’-NT signal levels correlate inversely with the degree of PrP knockout (i.e., the percentage of cells exhibiting the knockout). (G) Analysis of three separate ReN VM-derived PrP^-/-^ clones corroborates changes in 5’-NT expression relative to wild-type Ren VM cells.

When experimenting with CGs, we noticed that prolonged exposure (48 hrs) of undifferentiated ReN VM cells to non-toxic low nanomolar levels of ouabain caused the conspicuous appearance of a band that migrated in the denaturing SDS-PAGE with an apparent molecular mass of 60 kDa (**Fig 6A**). Alert to the possibility that the identity of this signal could serve as an endogenous surrogate reporter of NKA inhibition, we pursued its identification. To this end, we excised the band together with identically-sized control PVDF pieces from the same level of the blot but originating from the analysis of cells which had not been exposed to ouabain, undertook on-blot trypsinization, and LC-MS/MS analyses (**Fig 6B**). Queries of the human UniProt database with tandem mass spectra collected in each of a total six sample and control analyses revealed the signal to be derived from 5’-nucleotidase (5’-NT). This conclusion could be drawn on the basis of dozens of highly confident PSMs obtained for this protein (**Fig 6C**) and the fact that its presence or absence and relative abundance (approximated on the basis of its PSM counts) correlated well with the intensity of the corresponding band-of-interest in the Coomassie-stained blot membrane (**Fig 6D**). Next, this assignment was validated by immunoblotting of non-treated and ouabain treated samples with two 5’-NT-specific antibodies, which recognize non-overlapping epitopes within this protein. As expected, this analysis documented robust changes in the expression of 5’-NT following 48 hrs exposure of undifferentiated ReN VM cells to low nanomolar ouabain levels (**Fig 6E**). In parallel to these analyses, it had come to our attention that a band of the same apparent molecular mass was also robustly changed in Coomassie analyses of pools of ReN VM cells, in which the PrP gene had been targeted by two distinct CRISPR-Cas9 knockout gRNAs. Moreover, the increase in signal intensity for this band correlated inversely with the steady-state levels of PrP in these samples. 5’-NT-directed immunoblot analyses of the respective fractions pointed toward this signal to indeed also comprise 5’-NT (**Fig 6, panels F and G**). These results are consistent with the prior conclusion that PrP-deficiency partially impairs NKA activity and, as such, acts as a molecular mimic of cellular exposure to CGs.

### Exposure of ReN VM cells to CGs increases levels of GFAP and its calpain-dependent fragments

**Fig 7:**
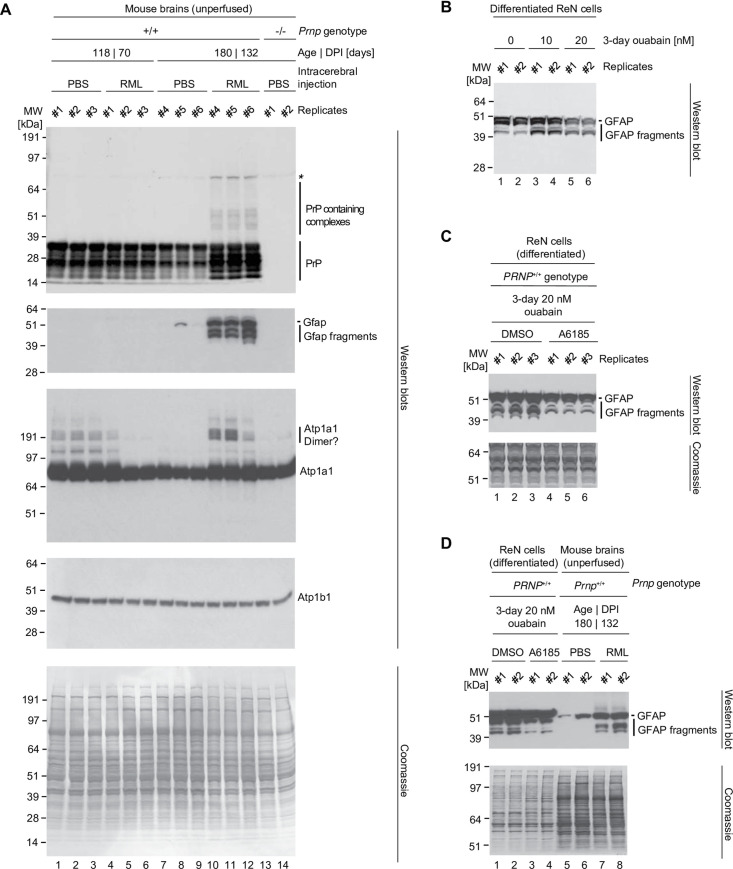
Prolonged exposure of ReN cells to cardiac glycoside or prion infection causes cleavage of GFAP. (A) Western blot documenting an increase in the intensity of GFAP antibody-reactive signals of 40–48 kDa in aged prion-infected mice that is not observed in vehicle-infected littermates. (B) Western blot documenting similar appearance of GFAP reactive bands in differentiated ReN cells exposed to low nanomolar concentrations of ouabain. (C) Calpain I inhibitor blocks ouabain-dependent formation of GFAP antibody reactive bands of 40–48 kDa. (D) Side-by-side western blot analysis of differentiated ReN cells, treated in the presence or absence of a calpain I inhibitor (A6185) next to brain lysates of RML-infected mice.

To begin to assess if partial inhibition of NKAs may play a role in prion diseases, we exploited the fact that ReN VM cells represent multi-potent neural stem cells, thereby providing a paradigm suitable for observing shifts in the differentiation of the main lineages of brain cells. Aside from the conversion of the prion protein that is central to the etiology, one hallmark shared amongst prion diseases is a profound astrogliosis. At the molecular level, the latter is characterized by an increase in the expression of the glial fibrillary acidic protein (GFAP) and the appearance of smaller bands that are reactive to GFAP-directed antibodies [21]. To assess a possible role of NKAs in this molecular phenotype, we infected mice with RML prions and investigated the effect of prion disease on levels of Gfap and NKAs. This analysis revealed the expected increase in Gfap levels in six months old mice that had been RML-infected and were sacrificed at 132 days post inoculation (DPI). Intriguingly, the increase in Gfap levels was paralleled by an increase in the levels of Atp1a1 antibody-reactive bands. This increase was most strikingly observed at the level of high molecular weight bands that possibly represented dimers or SDS-stable complexes (**Fig 7A, lanes 10–12**). Although bands migrating slower than the main Atp1a1 signal at around 90kDa were also seen in younger mice, the older PBS-inoculated age-matched control mice only exhibited very weak high molecular weight signals (**Fig 7A, lanes 7–9**). Next, we investigated if exposure of differentiated ReN VM cells to low levels of ouabain can mimic the GFAP cleavage *in vitro*. Indeed, three days following addition of nanomolar levels of ouabain to the cell culture medium, we observed an increase in the intensity levels of bands that were reactive toward GFAP-directed antibodies and migrated faster than the prominent GFAP signal at 50 kDa, i.e., with apparent molecular weights of around 40–45 kDa (**Fig 7B**).

One of the best-characterized consequences of CG inhibition of NKAs is an increase in intracellular calcium levels, which is mediated by the ensuing increase in intracellular Na^+^ concentrations driving an accumulation of intracellular Ca^2+^ levels through the activity of nearby Na,Ca-exchangers operating in reverse mode [22]. In fact, this phenomenon forms the basis for the CG-centered treatment of heart diseases that rely on the fortification of the calcium-dependent contraction of heart muscles. Because a previous report had shown that GFAP can be a target of calcium-dependent calpain proteases [23], we hypothesized that the lower mass GFAP-antibody-reactive bands might represent endoproteolytic calpain cleavage products, a model consistent with a previously reported activation of calpain levels in the hippocampus of prion-infected mice [21]. To explore this concept experimentally, we added a membrane-permeable calpain inhibitor (N-acetyl-Leu-Leu-norleucinal, also known as A6185) to a subset of cell culture dishes that were exposed to ouabain (**Fig 7C**). A GFAP-directed antibody revealed that the concomitant addition of the calpain inhibitor blocked the excessive formation of faster migrating bands, consistent with an endoproteolytic mechanism of their formation. The side-by-side analysis of total cellular protein extracts from prion-infected brains and ouabain-treated ReN VM cells revealed considerable similarities in GFAP antibody-reactive endoproteolytic band patterns (**Fig 7D**). Taken together, these data implicated the Ca^2+^-dependent activation of calpain endoproteases in the ouabain-induced GFAP proteolysis and may indicate that a similar course of events underlies the GFAP cleavage observed in prion diseases.

### CG-induced changes to PrP^C^ levels correlate imperfectly with NKA α subunit levels

**Fig 8:**
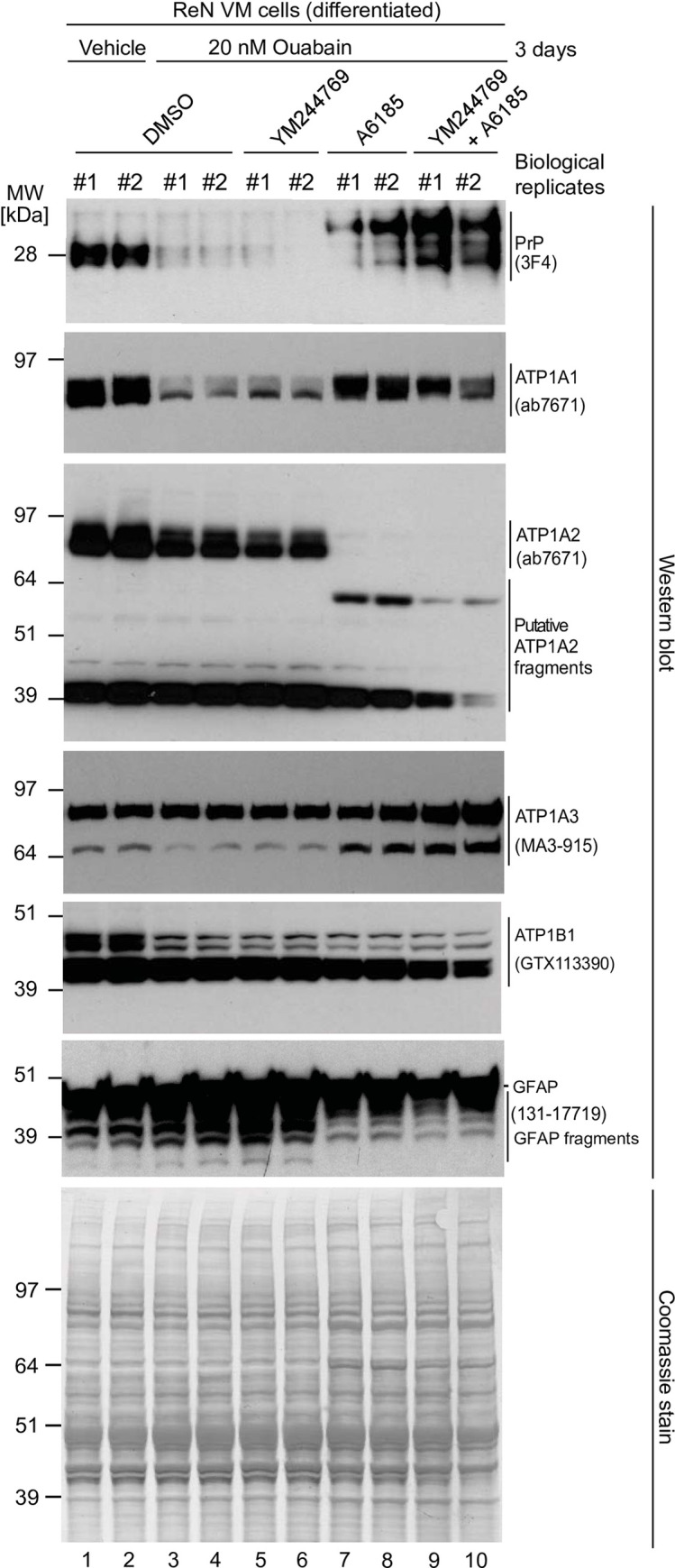
The steady-state levels of isoforms of PrP^C^ and NKA alpha-subunits are individually controlled by extracellular CGs and intracellular Ca^2+^ ions. Exposure to 20 nM ouabain caused a reduction in the steady-state levels of PrP^C^, ATP1A1 and ATP1A2—but not ATP1A3—in a manner that cannot be reversed by concomitant incubation with YM24476. In contrast, co-incubation of cells with ouabain and A6185 rescued the reduction in ATP1A1 levels and caused the appearance of signals detected with the PrP^C^- and ATP1A2-directed antibodies that migrated with different apparent molecular weights than the dominant signals for these proteins observed in untreated cells. Finally, the intensity of relatively fast migrating signals detected with a GFAP-directed antibody increase under conditions that favor intracellular calcium-dependent calpain activity.

The human genome encodes four NKA α subunits. Whereas expression of ATP1A4 is largely restricted to the testis [24], the expression of ATP1A1 is widespread, and ATP1A2 and ATP1A3 are observed in several tissues, including the brain. There, ATP1A3 is predominantly found in neurons, and ATP1A2 is abundant in astrocytes [25]. We next explored if CG exposure of ReN VM cells affects the steady-state levels of the three brain-expressed NKA α subunits equally. Moreover, to determine if any CG-effect on their expression levels depends on the action of the Na,Ca-exchanger, we repeated the CG-treatment of ReN VM cells in the presence or absence of a well-known inhibitor (YM24476) of the reverse mode activity of the Na,Ca-exchanger. Results from this experiment (**Fig 8**) corroborated the conclusion that steady-state levels of NKA subunits and PrP^C^, but not of a majority of other cellular proteins (see Coomassie stain), are strongly influenced by events that affect the NKA-mediated electrochemical gradient of Na^+^, K^+^ and Ca^2+^ ions. Intriguingly, this experiment revealed that changes to PrP^C^ expression levels that were observed in response to various treatment conditions corresponded closer to those observed for ATP1A1 and ATP1A2 than to ATP1A3. For instance, whereas steady-state levels of both ATP1A1 and ATP1A2 levels were reduced—together with PrP^C^ levels—in the presence of 20 nM ouabain, the most prominent western blot signal for ATP1A3 (and the main NKA beta subunit ATP1B1) were less affected by the prolonged exposure to this CG. Moreover, in cells whose NKA subunits were partially inhibited with ouabain, the additional blockade of calpain activity brought to the fore a slower migrating PrP antibody-reactive signal (**Fig 8, lanes 7–10**). The appearance of this signal was paralleled by a similarly striking effect on ATP1A2, whose canonical band migrating at around 90 kDa was almost entirely replaced with a faster migrating ATP1A2 antibody-reactive band at 60 kDa under this treatment condition. Finally, this experiment provided evidence that the ouabain-dependent reduction in steady-state PrP^C^ levels did not depend on the Na,Ca-exchanger acting in reverse mode, because blocking this activity of the channel by addition of YM24476 did not affect this outcome (**Fig 8**, compare PrP^C^ signals in **lanes 3 and 4** with those in **lanes 5 and 6**). Critically, the bulk of cellular proteins were not affected by these treatments as revealed by a consistent Coomassie staining pattern. Taken together, these data point toward a previously unappreciated immersion of PrP^C^ in cellular events that control the cellular electrochemical gradient.

## Discussion

Prompted by results from our recent work, which identified cell type-specific differences in the proteins that PrP^C^ binds to [9], we set out to revisit the molecular environment of PrP^C^ in the brain using refined tools and methodology. To our surprise, subunits of NKAs followed Ncam1 in this analysis as the top-ranked PrP^C^ interactors on the basis of both their profound co-enrichment and hundreds of PSMs assigned to them. In subsequent work, we initially validated that PrP^C^ and NKAs do indeed co-immunoprecipitate when captured from *in vivo* crosslinked brain extracts. Using the human ReN VM cell model we produced evidence of partial colocalization of PrP^C^ with ATP1A1 and established that cells respond to cardiac glycosides with changes to the steady-state levels of not just NKAs but also PrP^C^ in a manner that is surprisingly correlated. Next, we documented that the expression of PrP^C^ promotes NKA activity in a direct ion uptake assay. Corroborating these results, the knockout of PrP^C^ expression was observed to mimic a molecular phenotype of 5’-NT upregulation that can also be induced by partial inhibition of NKAs with CGs. Moreover, the cellular exposure to CGs can cause a calpain-dependent cleavage of the astrocytic intermediate filament protein GFAP that resembles a previously observed and here reproduced cleavage signature for this protein in prion-infected mice. Consistent with this interpretation, when inhibiting the calpain activation that occurs in the presence of CGs we prevented the GFAP cleavage. Taken together, our data reveal a complex interaction of NKAs and PrP^C^ that is strongly influenced by the NKA-dependent cellular ion homeostasis.

In our previous work we reported PrP^C^ to control NCAM1 polysialylation [26] and to be embedded in a specialized membrane domain enriched in molecules known to modulate TGF and integrin signaling in murine cell models [9]. Here we showed that murine brain PrP^C^, although still resident in proximity to NCAM1 and related cell adhesion molecules rich in Ig-like domains (Cntn1, Ncam2, L1cam, Nfasc, Cadm3, etc), is primarily surrounded by proteins that utilize ATP to generate ion gradients across cellular membranes (NKAs, Ca^2+^ ATPase, V-type H^+^ ATPase), or are part of the synaptic vesicle fusion apparatus (SYT1, SNAP25, SV2B) (**Fig 2**). It is currently unclear to which extent these differences in the molecular environment of PrP^C^ in cells versus brain reflect a reality of the brain being a highly specialized organ whose functioning relies on electrically mediated transmitter release or has more trivial explanations, namely the massive averaging of protein interactions that surely must occur when shifting an analysis of this kind from a cell to a tissue model. We suspect that both explanations have merit because it is well understood that NKAs are particularly abundant in the brain [27, 28]. On the other hand, it is also known that TGF beta and integrin signaling exists in the brain, but only a subset of brain cells will express high levels of these proteins, for example during neurogenesis and neuronal migration [29]. It is therefore plausible that their relative abundance in a PrP interactome will be diminished, unless cell type-specific interactors are studied [9]. Consistent with this interpretation, a majority of the latter PrP interactors were also enriched in proximity to PrP in this study but their overall levels were lower than in cell-based analyses (**S1 Table**). Importantly, the prominent spatial relationship to NKAs raises intriguing questions about PrP’s influence on the electrochemical gradient controlled by these pumps. Moreover, the possibility that PrP^C^ may also influence signaling emanating from these pumps, and that such influence may be perturbed in prion diseases raises the specter that NKAs could be mediators of a gain-of-toxic function widely attributed to PrP^Sc^.

The current study is not the first to link PrP^C^ physically to NKAs. In fact, at least five previous reports, including three from members of this team, had identified one or more subunits of NKAs in proximity to PrP^C^. Initially, we reported in 2004 that NKA subunits were observed in a PrP^C^ mouse brain interactome analysis [14]. Subsequently, the alpha-2 NKA subunit was identified as a PrP^C^ candidate interactor in a mouse brain-derived preparation of GFAP-decorated microsomes [30]. In hindsight, by choosing to restrict the analysis to GFAP-enriched microsomes, the authors may have biased the analysis in a way that favored the identification of the alpha-2 subunit. The latter is the primary NKA alpha paralog in astrocytes [28] but may—according to our current *in vivo* crosslinking data—just be one of three alpha-subunits that PrP^C^ resides next to in the brain. Moreover, the narrow focus on GFAP-enriched microsomes and a methodology that relied on the mass spectrometry-based identification of a few Coomassie-stained affinity-capture eluate bands, as opposed to a comprehensive discovery approach from whole brain material, masked the ability to assess the degree to which the alpha-2 subunit, as opposed to other interactors, resides in proximity to PrP^C^.

A less targeted approach by this group reported two other NKA subunits, Atp1a1 and Atp1b3, in a list or approximately 50 candidate PrP^C^ interactors in the N2a mouse neuroblastoma cell model [31, 32] before our recent PrP^C^ interactome analyses documented co-immunoprecipitation of the NKA alpha-1 subunit with PrP^C^ in three of four mouse cell models tested [9]. Finally, a PrP^C^-related interactome analysis based on a truncated PrP^C^ bait that comprises the N-terminal flexible domain (residues 23–144) also led to the co-enrichment of all brain-resident NKA alpha subunits, along with the NKA beta-1 subunit [33].

Altogether, the interaction between NKAs and PrP^C^ can be considered well-validated. However, in light of the pronounced expression of NKAs in the brain, their presence in affinity capture lists, including from our own group, could merely reflect the association of NKA with many other proteins, a scenario that often leads to highly abundant proteins, including tubulin and actin, to populate candidate interactor lists. Thus, to date not sufficiently appreciated has been the degree to which PrP^C^ in the brain resides in proximity to NKAs. Although the data presented in this report do not strictly rule out indirect binding, the most parsimonious interpretation of this pronounced level of NKA enrichment, supported by the crosslinking data we provided, is that a subset of NKAs are, in fact, direct neighbors of PrP^C^.

At this time missing is information regarding the NKA-PrP^C^ binding interface. NKA alpha-subunits are well-characterized to contribute a multi-span transmembrane domain and large cytosolic domains to the heteromeric NKA complexes [34, 35]. On the basis of topology and structural considerations, one may therefore predict that the indispensable beta-1 subunit contributes to the PrP^C^ binding interface. Not only is this protein, like PrP^C^, facing the extracellular space but its fold is also structurally related to the immunoglobulin-like and fibronectin-like IIII domains observed in NCAM1 and other members of the immunoglobulin superfamily of proteins [34, 36], whose PrP^C^ binding has been mapped [10] and partially resolved by NMR analyses [37].

Our analyses of NKA-mediated ^86^Rb^+^ uptake in the PrP-deficient ReN VM cells revealed a surprisingly robust positive influence of PrP^C^ on this measure of NKA activity that was subsequently also observed in N2a cells. Clearly, more work is needed to understand the mechanism by which this PrP^C^ influence on ion uptake manifests. Although we observed no differences in NKA steady-state levels, our data can currently not discern direct versus indirect effects of PrP expression on NKA activity. Although a direct effect seems intuitive due to the spatial proximity between these proteins, indirect effects may have contributed to this outcome. The latter may, for example, play out if PrP^C^ deficiency affected the differentiation state of the respective cells, which had to undergo clonal selection, in a manner that translated into lower NKA pump activity. The observation that PrP^C^-deficiency also impacted the NKA-independent ^86^Rb^+^ uptake, albeit to a lesser degree, by itself is an indication that there is considerable complexity to the full interpretation of these data. The future use of cell models that are engineered to allow the rapid induction or silencing of PrP^C^ may be better suited to resolve these questions.

**Fig 9:**
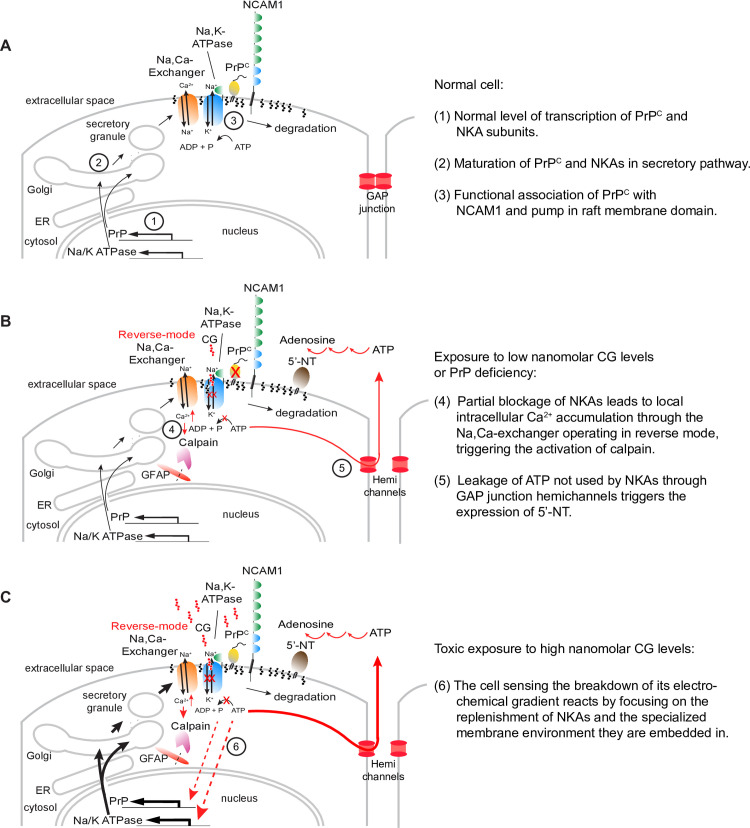
Cartoons depicting how PrP-knockout or CG exposure may cause 5-NT overexpression, GFAP cleavage and NKA overexpression. (A) Normal cell. (B) Cell exposed to low nanomolar CG levels or engineered to be PrP deficient. (C) Cell exposed to toxic high nanomolar CG levels. Note that the cartoon depicts a generic cell and omits subtleties related to the existence of NKA paralogs and isoforms.

Consistent with the interpretation that removal of PrP^C^ mimicked the addition of CGs, we also observed an upregulation of 5’-NT in PrP-deficient cells. An increase in the activity of ATP hydrolyzing ectonucleotidases has previously been described in hippocampal (but not in cortical) synaptosome preparations of *Prnp* null mice [38]. How does exposure of CGs or the reduction of PrP levels activate 5’-NT? Although the answer to this question has not been firmly established, one plausible scenario is as follows: according to our data, both events cause NKA inhibition. The latter is well known to promote the accumulation of intracellular Ca^2+^ through the passively operating Na^+^,Ca^2+^-exchanger that relies on the pump-generated Na^+^ gradient for exporting Ca^2+^ from the cytosol but operates in reverse mode when the normal electrochemical gradient has been compromised. Even low concentrations of ouabain (or other CGs), insufficient for blocking ion transport by the pump, have been reported to cause local Ca^2+^ increases, possibly through a signaling complex that involves the inositol 1,4,5-trisphosphate receptor (IP3R) [39]. Several authors have reported that changes in calcium levels transmit to neighboring cells, a phenomenon relying on gap junctional intercellular communication [40]. Others have reported that the detachment of gap junction hemichannels composed of connexins, which can be invoked by elevated calcium, leads to cellular ATP leakage into the extracellular space [41–43]. The spike in extracellular ATP would be expected to trigger the expression of 5’-NT [44], the final enzyme in an extracellular cascade of ATP dephosphorylation steps [45]. Consistent with this model (**Fig 9**), 5‘-NT plays a role in the response to vascular leakage [46] and serves as a marker of mesenchymal stem cells [47, 48], its expression being dramatically upregulated during epithelial-to-mesenchymal transition [26, 49–51], when cell-to-cell contacts are broken and cells move to integrin-mediated cell substrate adherence [52]. Interestingly, the expression of 5’-NT was also observed to directly correlate with astrogliosis [53], an observation that provides a segue to the second set of observations we made in this study, which cumulatively suggest that PrP^Sc^ may inhibit NKA uptake activity (**Fig 5F**)—despite an increase in total ATP1A1 signal intensity we observed in prion-infected ScN2a cells (**Fig 5E**). In light of the above, the gradual poisoning of NKAs by PrP^Sc^ could mimic CG exposure and contribute to the increase in GFAP that has repeatedly been observed in prion diseases, together with enhanced calpain cleavage [54, 55]. Notably, this calpain activation would not only produce GFAP cleavage products but may also downregulate PrP^C^, thereby removing the essential substrate for prion conversion and slowing the disease, a phenomenon proposed to underlie the extraordinary long incubation periods observed in prion diseases [56]. Moreover, proteins that share biochemical characteristics and raft-residence with PrP^C^ may also get depleted by the enhanced local calpain activity, accounting for their concomitant prion disease-associated depletion, previously reported for the PrP paralog Shadoo [57]. It is increasingly recognized that a prominent subset of NKAs associate with membrane rafts in a wide range of cell types. The presence of cholesterol and caveolin provides an environment that is conducive to the NKA ion pumping activity [58]. Moreover, NKAs were visualized in hot spots interpreted as raft domains in neurites of cerebellar granule cells [59] and were observed in the low-density fraction enriched in cholesterol and sphingolipids in renal epithelial cells [60]. One study estimated that 30–40% of Atp1a1 and 80–90% of Atp1b1 from rat cardiac myocytes segregate with caveolar membrane preparations, which comprised 75% of total myocyte NKA activity [61]. A chain of events, whereby PrP^Sc^ poisons and draws a subset of nearby NKAs into the insoluble complexes it forms, is consistent with a previous observation of NKAs in PrP^Sc^ preparations [62] and the complexity of Atp1a1 western blot signals we observed in the current study in RML-infected but not in mock-infected mouse brains.

## Conclusion

This study raised more questions than it could answer, leaving a need to connect the dots. Rather than proposing a novel function for PrP^C^, we anticipate that further work will reveal a complex reality of crosstalk between an ATP utilizing molecular biology that controls electrochemical membrane gradients and a cellular program guided by TGFB1 and integrins that controls a cell’s interactions with its environment. The little we know thus far points toward a previously unappreciated positive influence of PrP^C^ on the activity of NKAs and raises the specter of perturbed interactions with these ion pumps playing a role in the pathobiology of prion diseases.

## Material and methods

All experiments were unblinded and non-randomized. For all experiments, all technical replicates from all biological replicates were included in data analysis.

### Antibodies

Immunoaffinity capture made use of a humanized recombinant PrP-directed Fab, known as D18 that was generously provided by the laboratory of Dr. Emil F. Pai, University of Toronto.

For immunoblotting we used antibodies against PrP (clone 3F4, catalog number MAB1562, Millipore, ON, Canada, and clone Sha31, catalog number A03213, Bertin Bioreagent, Montigny le Bretonneux, France), ATP1A1 (catalog number ab7671; Abcam, ON, Canada), ATP1A2 (catalog number AB9094-I, Millipore, ON, Canada), ATP1A3 (catalog number MA3-915, Thermo Fisher Scientific, MA, USA), ATP1B1 (catalog number GTX113390; GeneTex, CA, USA), 5’-NT (catalog number HPA17357, Sigma-Aldrich, ON, Canada, and catalog number AB175396, Abcam), GFAP (catalog number 131–17719, Thermo Fisher Scientific), and ACTB (catalog number sc-47778, Santa Cruz Biotechnology, TX, USA).

The immunocytochemistry analyses employed a distinct ATP1A1-directed antibody (catalog number 14418-1-AP, Proteintech, IL, USA) along with anti-PrP (clone 3F4, see above) as well as secondary goat anti-rabbit antibody 647 (catalog number A-21244, Invitrogen, ON, Canada) and goat anti-mouse antibody 488 (catalog number A-11029, Invitrogen, ON, Canada).

### Mice

Twenty-one mice were used in total. Each experimental unit was one mouse (biological replicate). *A priori* study criteria for exclusion of any study animal included deviation from typical growth or health profiles of their respective line, as assessed by University Hospital Network veterinary staff. The study made use of a previously described FVB/Ncrl *Prnp* null mouse line [5] that was generously shared with us by Dr. David Westaway, University of Alberta, and was in-house bred as hemizygous *Prnp*^+/-^ mice. CD1 outbred mice were sourced from a commercial mouse repository (JAX, Maine, USA) and were used for the prion inoculation experiments. During the animal studies, mice were housed in comparable environments in the same room to minimize potential confounders. The mice were on an artificial 12-hour day/night cycle in microisolator cages kept at elevated room temperature (24°C) with no more than five mice per cage. Feed was 18% protein chow and acidified water offered *ad libitum*. The mice underwent daily health checks for general appearance and activity, and their cages were changed once a week. No behavioral studies were undertaken, and the mice were aged exclusively for the collection of brain samples. Mice were euthanized through deep anesthesia by inhalation of 5% isoflurane at 1.5 liters/min of oxygen. All animal procedures were in accordance with the Canadian Council on Animal Care, reviewed and authorized by the University Health Network Animal Care Committee and approved under Animal Use Protocol 6182.

### PrP interactome analyses

Immunoprecipitation of PrP was performed from time-controlled transcardiac perfusion crosslinked mouse brains [14] using a methodology described in detail previously [13]. The crosslinked brains were homogenized by bead beating in 150 mM Tris-HCl pH 8.3, 150 mM NaCl, 0.5% (v/v) NP-40 and 0.5% sodium deoxycholate supplemented with protease and phosphatase inhibitor cocktails (11836170001 and 4906837001; Millipore). Brain homogenates were diluted to equal total protein concentration. The PrP-directed affinity-capture made use of the recombinant D18 antibody conjugated to KappaSelect beads (17-5458-01; GE Healthcare, ON, Canada). The immunoprecipitation media was extensively washed then treated with 20% acetonitrile in aqueous 0.2% trifluoroacetic acid (v/v) to recover bound protein [9]. After drying by centrifugal concentration, the eluates were denatured in 9 M urea then reacted with tris(2-carboxyethyl)phosphene (TCEP) at pH 8 and 60°C for 30 minutes in 500 mM triethyl ammonium bicarbonate (TEAB) buffer. Upon cooling, the lysates were reacted with 4-vinylpyridine for one hour then digested with trypsin in 500 mM TEAB overnight at 37°C. Trypsin digests were reacted with iTRAQ reagents (Sciex, Concord, ON, Canada) according to the manufacturer’s instructions then combined and purified on C18 microcolumns (Agilent, Santa Clara, CA, USA).

### Mass spectrometry and protein sequencing

The affinity capture of PrP^C^ and its crosslinked molecular neighbors made use of a recombinant humanized antibody (D18) known to target a non-linear epitope comprising PrP residues 133–157. The sample handling of co-immunoprecipitates included stringent washing of affinity capture matrices to minimize non-specific binding [63]. Raw data for peptide sequencing (MS and MS^2^) and quantification (synchronous precursor selection MS^3^) were acquired on an Easy-nLC 1000-Orbitrap Fusion platform. Chromatographic separation was achieved using a binary acetonitrile/water gradient operated for one (polyacrylamide gel and PVDF bands) or four (immunoprecipitates) hours. The mobile phase went from 0 to 30% acetonitrile over the first two thirds of each HPLC run, then to 100% acetonitrile for the final 10% of the run time. Proteome Discoverer 1.4 software was used for protein sequencing by Mascot and Sequest HT algorithms. The false discovery rate analysis was conducted, and mass spectra were stringently filtered using the ‘Percolator’ discriminator routine within ProteomeDiscoverer (version 1.4.0.288) and matched to entries in the mouse UniProt database comprising canonical and isoform sequences (version Aug 21, 2019; downloaded on Nov 8, 2019).

### Cell culture

ReN VM neural progenitor (catalog number SCC008) and Neuro-2a mouse neuroblast (catalog number CCL-131) cells were obtained from Millipore and ATCC (VA, USA), respectively. The undifferentiated ReN VM cells were grown on Matrigel basement membrane matrix (catalog number 354230, Corning, NY, USA) and maintained in DMEM/F12 (catalog number 11320033, Thermo Fisher Scientific) supplemented with 20ng/ml basic fibroblast growth factor (catalog number 8910, Cell Signaling, MA, USA), 20ng/ml epidermal growth factor (catalog number RKP01133, Reprokine, FL, USA), 10 Units/ml heparin sodium salt (catalog number H3149, Sigma-Aldrich) and 1X N21-MAX (catalog number AR008, R&D Systems, MN, USA) or 1X B27 media supplement (catalog number 17504044, Thermo Fisher Scientific). The cells were differentiated into neuronal and glial populations with the removal of heparin and growth factors from media for at least 7 days. Passage-matched N2a and RML-infected N2a cells were created as described previously [64] and maintained in DMEM (catalog number 11995065, Thermo Fisher Scientific), supplemented with 10% fetal bovine serum (catalog number 12483020, Thermo Fisher Scientific) and 1% GlutaMAX (catalog number 35050061, Thermo Fisher Scientific). CRISPR-Cas9-generated N2a knockout cells were described previously [65].

### Immunocytochemistry

ReN VM cells were cultured on glass coverslips according to the procedure described above. Following 7 days of differentiation, cells were fixed with 4% paraformaldehyde (w/v in PBS), followed by incubation with permeabilization and blocking buffer (0.1% Triton X-100 (v/v), 1% BSA (w/v) in PBS). Cells were incubated overnight at 4°C with the PrP-directed and ATP1A1-directed antibodies added at a 1:100 dilution. Upon removal of the primary antibodies, cells were incubated at 1:400 dilutions for 90 minutes at ambient temperature with secondary antibodies conjugated to fluorescent dies that emit at 647 and 488 nm wavelength for the detection of ATP1A1 and PrP, respectively. Next, cover slips were mounted onto glass slides using ProLong Gold with DAPI antifade mounting agent. Deconvolution was undertaken and the degree of co-localization was computed using built-in algorithms within the ‘Zen black’ microscopy analysis software (version 8.1.0.484, Zeiss Microscopy, Jena, Germany).

### CRISPR/Cas9 knockout of PrP in ReN cells

The methodology to develop knockout clones was as described previously [65]. Briefly, the selected gRNA human PrP (CACTGGGGGCAGCCGATACCCGG) was cloned into the gRNA plasmid (catalog number MLM3636, Addgene, MA, USA) following digestion with the BsmBI enzyme (catalog number R0580S, New England BioLabs, MA, USA) and verified by Sanger sequencing. The gRNA was then transfected into the previously transduced ReN VM cells with the Cas9 enzyme using Lipofectamine Stem Transfection reagent (catalog number GST-2174, Thermo Fisher Scientific). After 48 hours, cells were diluted and grown to form single cell-based colonies and screened for PrP expression by immunoblotting.

### ^86^Rb^+^ uptake assay

Cells were grown in 24-well format plates to 90% confluency. Media were changed to plain DMEM-F12 three hours before the ^86^Rb^+^ uptake measurements. Each condition was analyzed in the presence and absence of 1 mM ouabain and in three biological replicates. Following the addition of 2μCi ^86^Rb^+^ (catalog number NEZ072, Perkin Elmer, MA, USA) per well, cells were incubated at 37°C for 10 minutes and then the uptake stopped by 4 cold washes of 100mM MgCl_2_ and 10mM HEPES-Tris buffer (pH 7.4). Cells were then lysed in 1% NP40, 150mM NaCl and 150mM Tris (pH 8.3). Following the measurement of total protein content using BCA assay, identical 5uL aliquots of the lysates were diluted in 1ml water and the measurement of their radioactivity in a scintillation counter (Perkin Elmer) capitalized on the Cherenkov radiation generated in water by the emission of high-energy ^86^Rb^+^ radiation. The fourth wash solution was observed to give rise to negligible counts per minute (cpm) than the lysates (approximately 10,000-fold lower), suggesting that the vast majority of ^86^Rb^+^ signal was indeed cell-associated. A calibration curve for the translation of cpm values into molar amounts of ^86^Rb^+^ was obtained by generating a dilution series of the commercial ^86^Rb^+^ stock of known specific activity in water. The ^86^Rb^+^ uptake is reported in nmol Rb uptake / min / mg protein.

### Western blot analyses

In order to harvest cultured cells, they were washed with cold PBS once and then, lysed in cold lysis buffer containing 150mM NaCl, 150mM Tris-HCl (pH 8.3), 1% NP40 plus protease and phosphatase inhibitors. Brain homogenates were created in lysis buffer containing 150mM NaCl, 150mM Tris-HCl (pH 8.3), 0.5% NP40 and 0.5% sodium deoxycholate supplemented by protease and phosphatase inhibitors. In both cases, samples were spun down to remove the insoluble debris and protein concentration was determined using bicinchoninic acid (BCA) colorimetric protein assay kit (catalog number 23225, Thermo Fisher Scientific). For samples whose SDS-PAGE analyses was preceded by the enzymatic removal of N-glycans, the PNGase F digestion followed the detailed protocol provided by the manufacturer (catalog number P0704S, New England Biolabs). Equal amounts of total protein were loaded and run on Bis-Tris denaturing gels (catalog number NW04125BOX, Thermo Fisher Scientific) and transferred to Polyvinylidene fluoride membranes (PVDF, catalog number IPVH00010, Millipore). The membranes were blocked in 10% skim-milk (in TBS-T) and probed with the primary antibodies overnight at 4°C. The next day the blots were washed in TBS-tween, three times and incubated with the relevant secondary antibodies (catalog numbers 7074S and 7076S, Cell Signaling Technologies, MA, USA) for an hour at room temperature followed by three times of TBS-tween wash to remove the unbound antibodies. The signals were development using either X-ray films or a LI-COR Odyssey Fc digital imaging system (LI-COR Biosciences, NE, USA).

### Inhibitor treatments of ReN VM cells

Ouabain octahydrate (catalog number O3125) and Calpain Inhibitor I (catalog number A6185) were purchased from Sigma-Aldrich and dissolved in water and DMSO respectively to generate stock solutions with 1000 X working concentrations prior to the treatment. YM244769 was sourced from TOCRIS (catalog number 4544) and dissolved in DMSO. The treatments were renewed daily for the indicated duration.

When specified, differentiated ReN VM cells were treated with CGs for 3 or 7 consecutive days with daily renewal of half of differentiation media plus the inhibitors. When applicable, calpain inhibitor I or YM244769 were added 2h before the initial ouabain treatment at a final concentration of 20 μM and replenished daily along with half media changes on differentiated ReN VM cells.

### RT-qPCR analyses

To quantify ATP1A1 and PRNP transcript levels in ReN VM cells following vehicle or Ouabain treatment at 10nM, 30nM and 50nM conditions, cells were treated every other day over a period of 7 days in biological triplicates and qPCR was conducted in technical duplicates, each reaction containing 50 ng of cDNA, TaqMan master mix (catalog number 4427788, Thermo Fisher) and one of the following TaqMan assay kits (Thermo Fisher) targeting of human PRNP (catalog number Hs01920617_s1), ATP1A1 (catalog number Hs00933601_m1), ATP1A2 (catalog number Hs01560076_g1) or ATP1A3 (catalog number Hs00958036_m1). Transcript abundance was normalized using the reference genes PPIA (catalog number Hs99999904_m1) and GAPDH (catalog number (Hs03929097_g1). Negative controls included template controls for each gene expression assay along with a no probe control that all produced no signal of transcript amplification.

### Identification of ouabain-dependent 5’-NT upregulation

Lysates from ouabain-treated and mock-treated ReN VM cell cultures were subjected to SDS-PAGE and western blot analyses. From lanes containing the ouabain-treated and mock-treated samples, Coomassie blue-stained bands of approximately 60 KDa were excised and destained with 50% acetonitrile in 100 mM NH_4_HCO_3_ (polyacrylamide) or 25% acetic acid in 50% methanol (PVDF). Polyacrylamide bands were stored at 60°C in 10 mM dithiothreitol for 15 minutes, then in the dark for 20 minutes in 15 mM iodoacetamide. PVDF bands were treated with 0.5% polyvinylpyrrolidone (w/v) in 100 mM acetic acid at 37°C with shaking at 300 rpm for 1 hour, then washed with water. The bands were each covered in 125 ng of trypsin (Promega, Madison, WI, USA) in 100 mM NH_4_HCO_3_ at 37°C overnight and the resulting peptides were extracted with 50% acetonitrile (v/v) in 100 mM NH_4_HCO_3_. The extracts were dried in a centrifugal concentrator, resuspended in 0.1% formic acid (v/v), then concentrated on C18 microcolumns (Agilent, Santa Clara, CA, USA) and analyzed by LC-MS/MS.

Proteins sequenced from polyacrylamide or PVDF bands were first sorted according to the number of spectral counts by which they were identified from ouabain-treated samples and second by the ratio of peptide-to-spectral matches in ouabain-treated samples, relative to mock-treated samples. 5’-NT (Uniprot accession number P21589-2) was the most enriched protein in ouabain-treated samples based on this ranking.

### Analysis of prion-infected mouse brains

CD1 mice were intracerebrally inoculated into the left hemisphere using free-hand technique with 10 μL of 1:10 brain homogenate diluted in PBS (v/v). This homogenate was sourced from a mouse that had succumbed to prion disease following its own injection with Rocky Mountain Laboratory (RML) prions. As negative controls served CD1 mice inoculated in an identical manner with 10 μL of PBS. 70 days or 132 days post-inoculation, the respective cohorts of mice were subjected to transcardiac perfusion with PBS.

### Statistics

The statistical comparison of treatment cohorts was based on three biological replicates per cohort and assumed that independent sample sets were of unknown variance. Accordingly, the computation of *p*-values was based on the two-tailed t-test. Following convention, a single asterisk designates a *p*-value < 0.05, for each additional asterisk shown a tenfold lower *p*-value threshold was met.

## Supporting information

S1 Fig Comparison of PrP-specific interactors and non-specific binders. (A) Graph comparing cross-correlation values of proteins interpreted to represent non-specific (protein levels WT/KO <2) versus specific PrP interactors (protein levels WT/KO ≥2) on the basis of their association with wild-type versus PrP-knockout co-IP samples. Distributions were normalized and represent Sequest HT Xcorr values. (B) Chart depicting the normalized distribution of enrichment ratios (wildtype/knockout) of the non-specific or specific PrP interactors shown in Panel A. (C) Selective enrichment of Ncam1-derived peptides in PrP-co-immunoprecipitation eluates derived wild-type brains but not in PrP ko eluates. See legend to **Fig 2** for graphing details.
(PDF)

S2 Fig Prolonged exposure of ReN VM cells to 50 nM Ouabain causes internalization of full-length PrP^C^ protein and transcriptional activation of the PRNP gene. (A, B) Representative immunocytochemical analyses of cells treated for seven days with vehicle or 16 nM Ouabain. Note the partial co-localization of PrP^C^ and ATP1A1 at the cell surface and in intracellular vesicular structures. (C) No signals were detected when PrP-deficient ReN VM cells were exposed to the PrP^C^-reactive antibody 3F4 and the secondary AF633-conjugated antibody. Similarly, exposure of cells to secondary antibodies alone did not give rise to signals (not shown). (D) The predominant post-translational isoform of steady-state PrP^C^ levels in ReN VM cells is the full-length protein and this does not change when the cells are exposed for seven days to 10, 30 and 50 nM concentrations of Ouabain. Accordingly, enzymatic removal of N-glycans with PNGase F reveals the main PrP^C^-reactive band to migrate with an apparent molecular weight of approximately 28 kDa. (E, F) Cells exposed for seven days to 50 nM Ouabain concentrations react by increasing their ATP1A1 and PRNP transcript levels. mRNA levels differed significantly between the 0 nM and 50 nM conditions for ATP1A1 (p = 0.029) and PRNP (p = 0.003) assays (paired two-tailed t-test). Error bars represent the standard deviation for six replicates.
(PDF)

S1 Table PrP interactors in mouse brain (full list; sorted on the basis of relative PrP co-enrichment; includes nonspecific interactors). Levels of the tubulin alpha 1 subunit (Tuba1a), a protein we considered a non-specific interactor, were used to normalize enrichment levels following the assumption that differences in bulk surface in wild-type affinity matrices would skew relative binding of non-specific matrix interactors. Proteins preceding Tuba1a in this table exhibited PrP co-enrichment, with highest ranked proteins representing strong PrP candidate interactors.
(PDF)

S1 Raw images Compilation of uncropped western blot and gel images. (PDF)

## Abbreviations

5’-NT, 5’: nucleotidase
CG: cardiac glycoside
CRISPR: clustered regularly interspaced short palindromic repeats
DPI: days post inoculation
EMT: epithelial-to-mesenchymal transition
GFAP: glial fibrillary acidic protein
iTRAQ: isobaric tag for relative and absolute quantitation
IP: immunoaffinity purification
MS/MS: tandem mass spectrometry
NKA: Na,K-ATPase
PrP: prion protein
PrP^C^: cellular prion protein
PSM: peptide-to-spectrum match
RT-qPCR: reverse transcription quantitative polymerase chain reaction
