# Supplementary figures and images for: The cellular prion protein interacts with and promotes the activity of Na,K-ATPases
Source: PLoS One. 2021 Nov 30;16(11):e0258682. doi: 10.1371/journal.pone.0258682 (PMC8631662; doi:10.1371/journal.pone.0258682)

S1 Figure

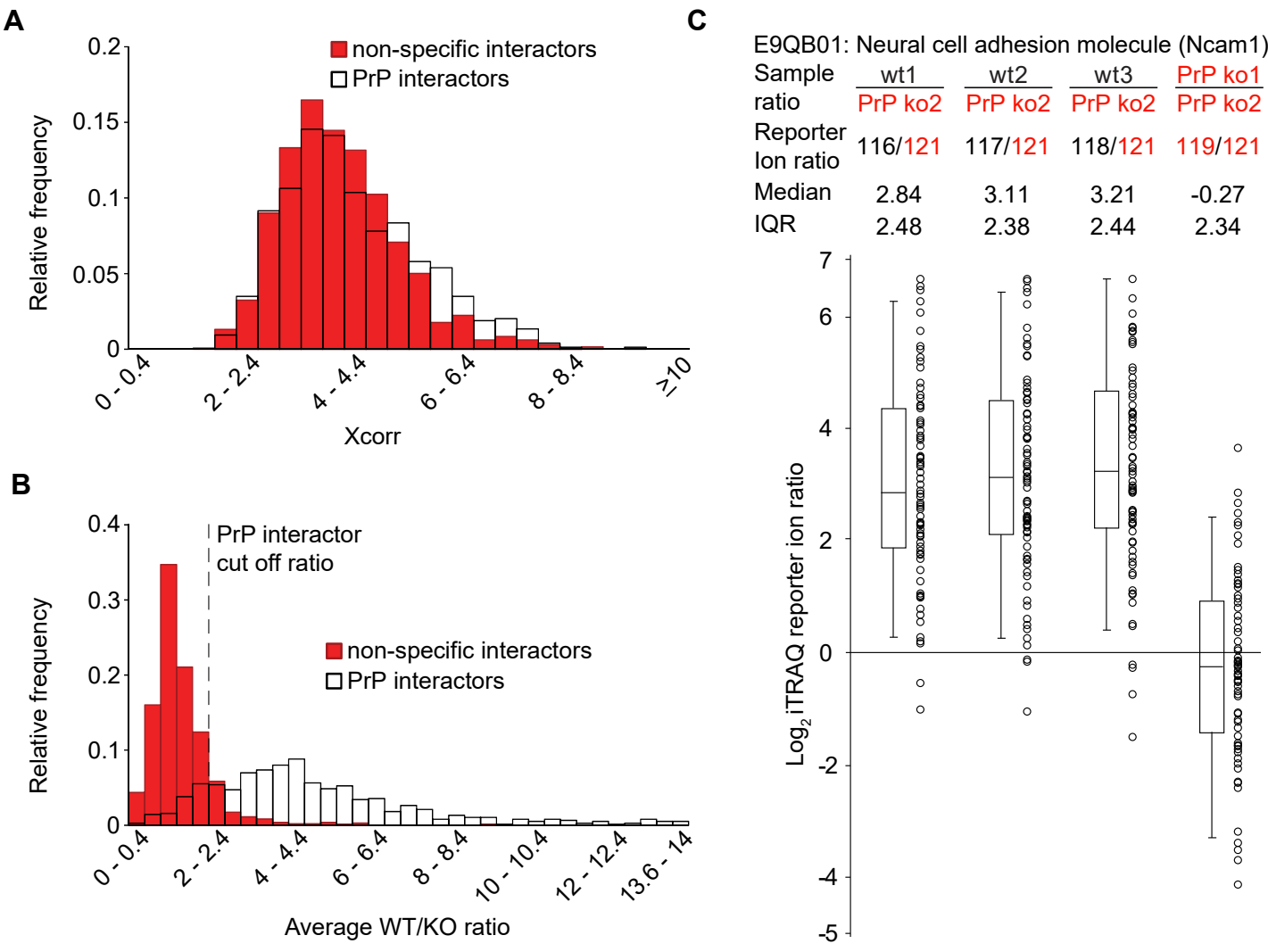

Supplement: S1 Fig — (A) Graph comparing cross-correlation values of proteins interpreted to represent non-specific (protein levels WT/KO <2) versus specific PrP interactors (protein levels WT/KO ≥2) on the basis of their association with wild-type versus PrP-knockout co-IP samples. Distributions were normalized and represent Sequest HT Xcorr values. (B) Chart depicting the normalized distribution of enrichment ratios (wildtype/knockout) of the non-specific or specific PrP interactors shown in Panel A. (C) Selective enrichment of Ncam1-derived peptides in PrP-co-immunoprecipitation eluates derived wild-type brains but not in PrP ko eluates. See legend to Fig 2 for graphing details. (PDF) [file pone.0258682.s001.pdf]

S2 Figure

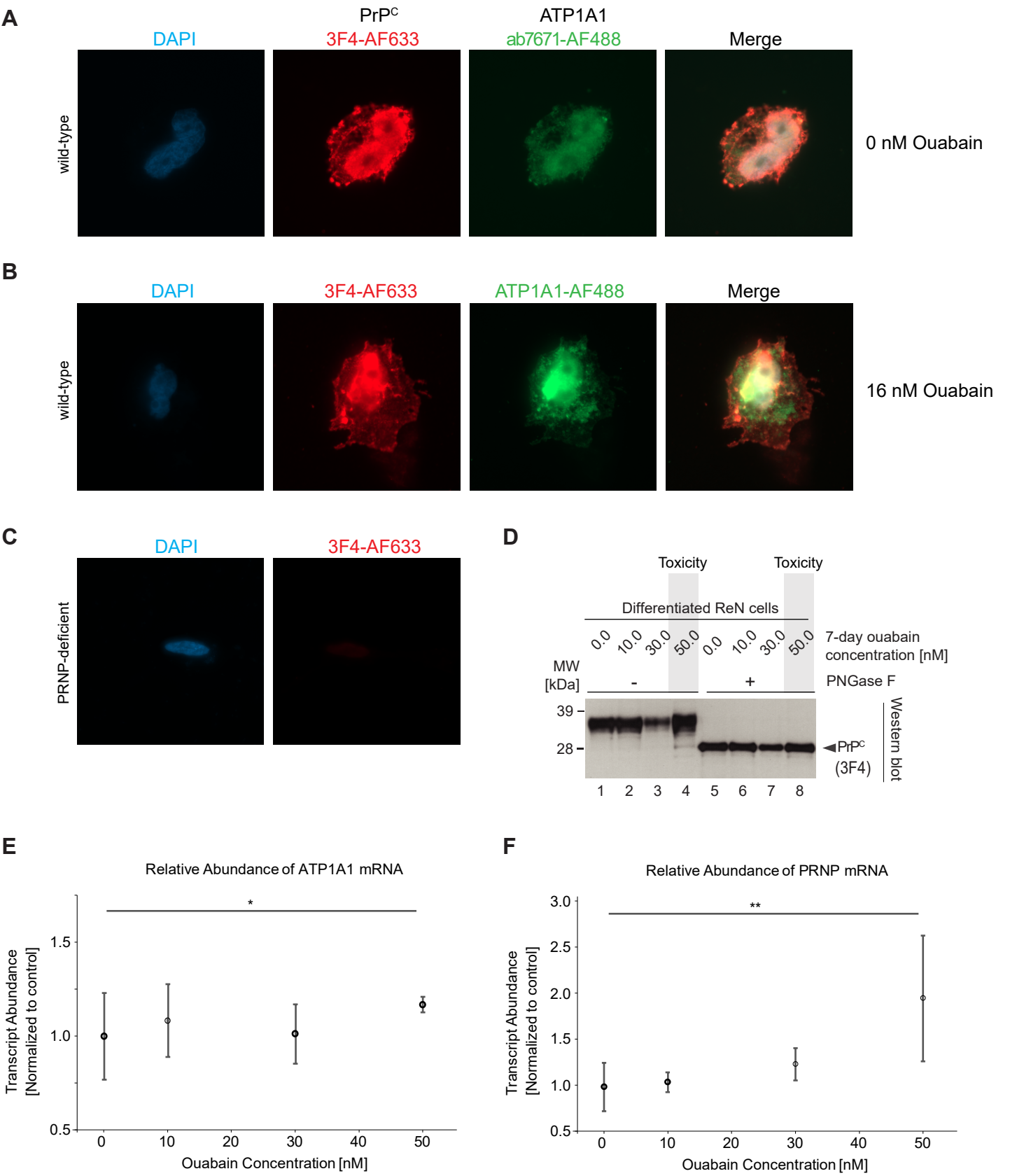

Supplement: S2 Fig — (A, B) Representative immunocytochemical analyses of cells treated for seven days with vehicle or 16 nM Ouabain. Note the partial co-localization of PrPC and ATP1A1 at the cell surface and in intracellular vesicular structures. (C) No signals were detected when PrP-deficient ReN VM cells were exposed to the PrPC-reactive antibody 3F4 and the secondary AF633-conjugated antibody. Similarly, exposure of cells to secondary antibodies alone did not give rise to signals (not shown). (D) The predominant post-translational isoform of steady-state PrPC levels in ReN VM cells is the full-length protein and this does not change when the cells are exposed for seven days to 10, 30 and 50 nM concentrations of Ouabain. Accordingly, enzymatic removal of N-glycans with PNGase F reveals the main PrPC-reactive band to migrate with an apparent molecular weight of approximately 28 kDa. (E, F) Cells exposed for seven days to 50 nM Ouabain concentrations react by increasing their ATP1A1 and PRNP transcript levels. mRNA levels differed significantly between the 0 nM and 50 nM conditions for ATP1A1 (p = 0.029) and PRNP (p = 0.003) assays (paired two-tailed t-test). Error bars represent the standard deviation for six replicates. (PDF) [file pone.0258682.s002.pdf]

S1\_raw\_images

Figure 4

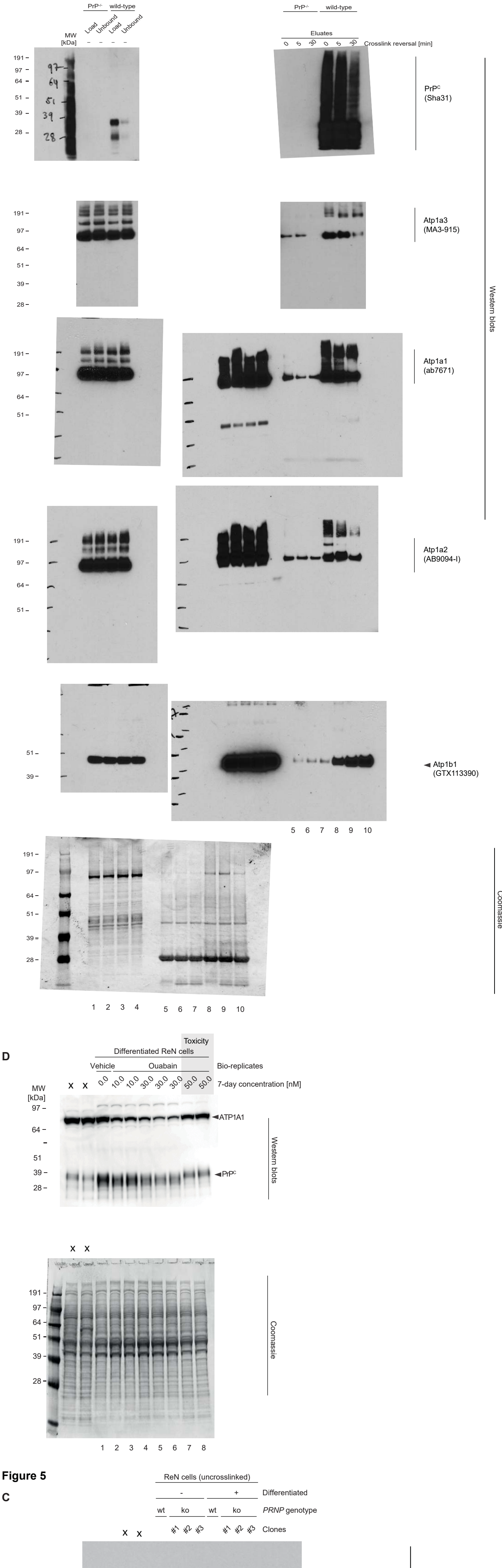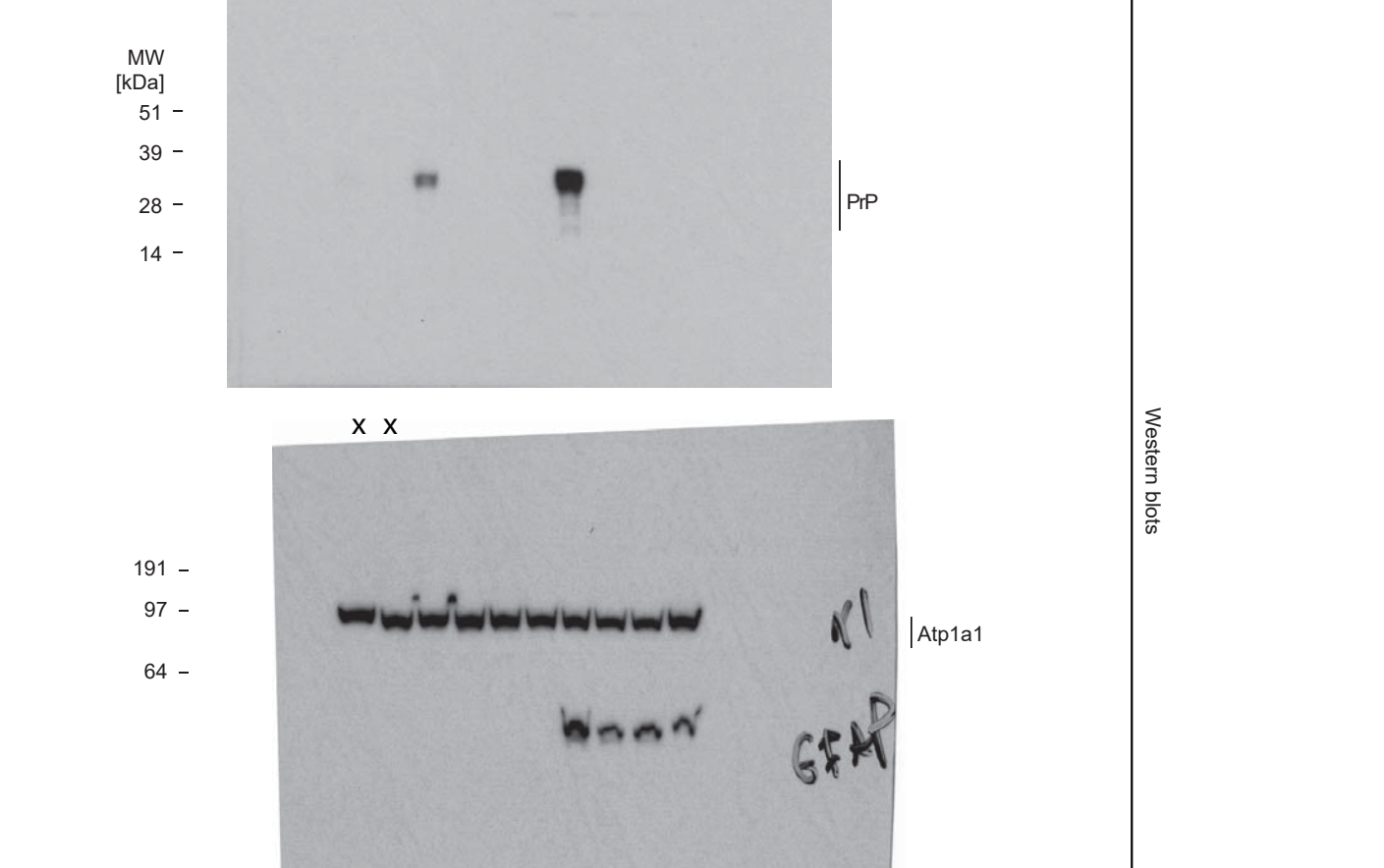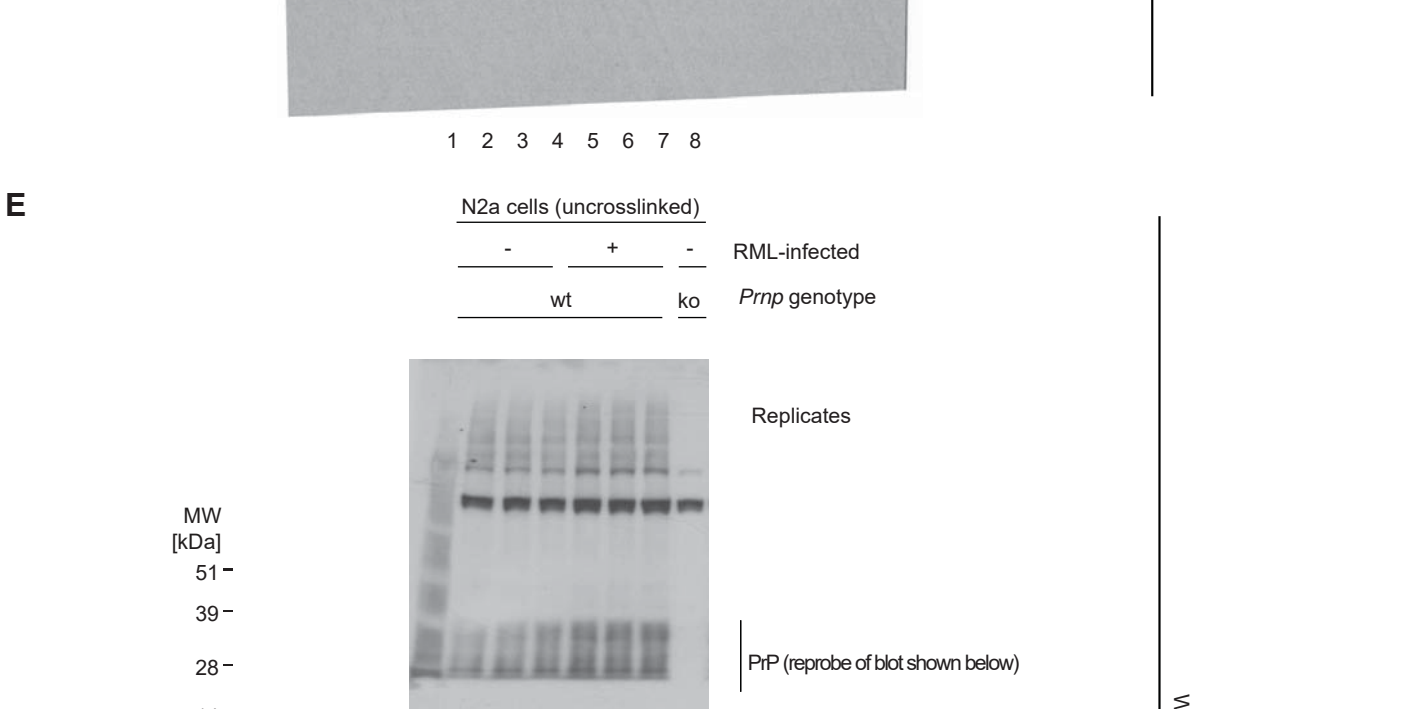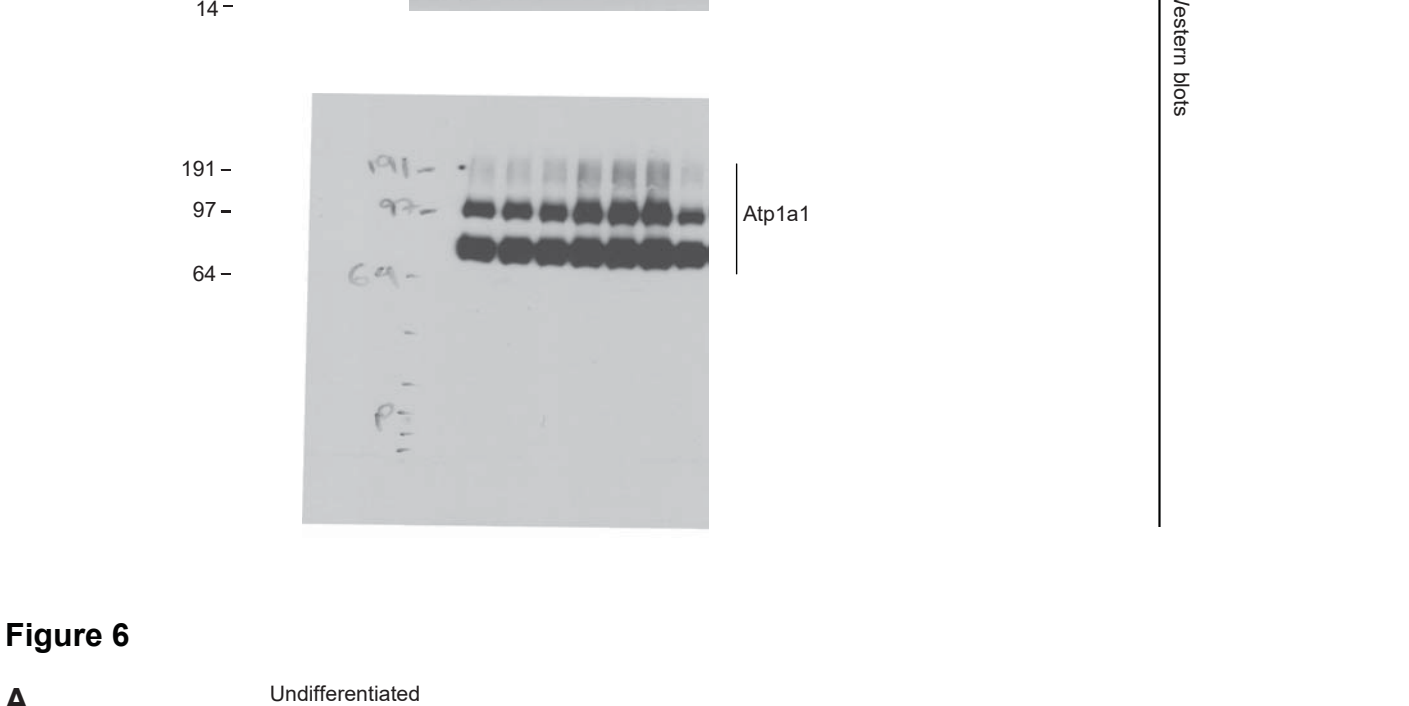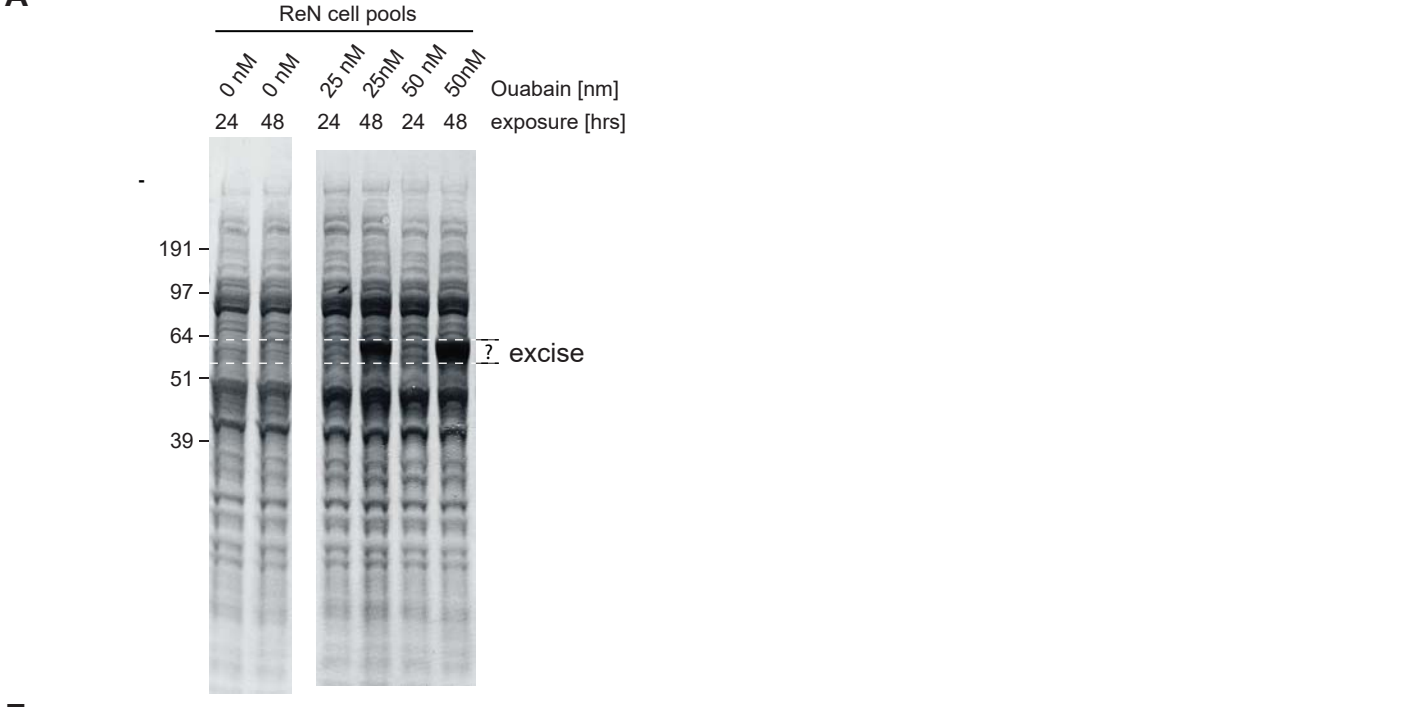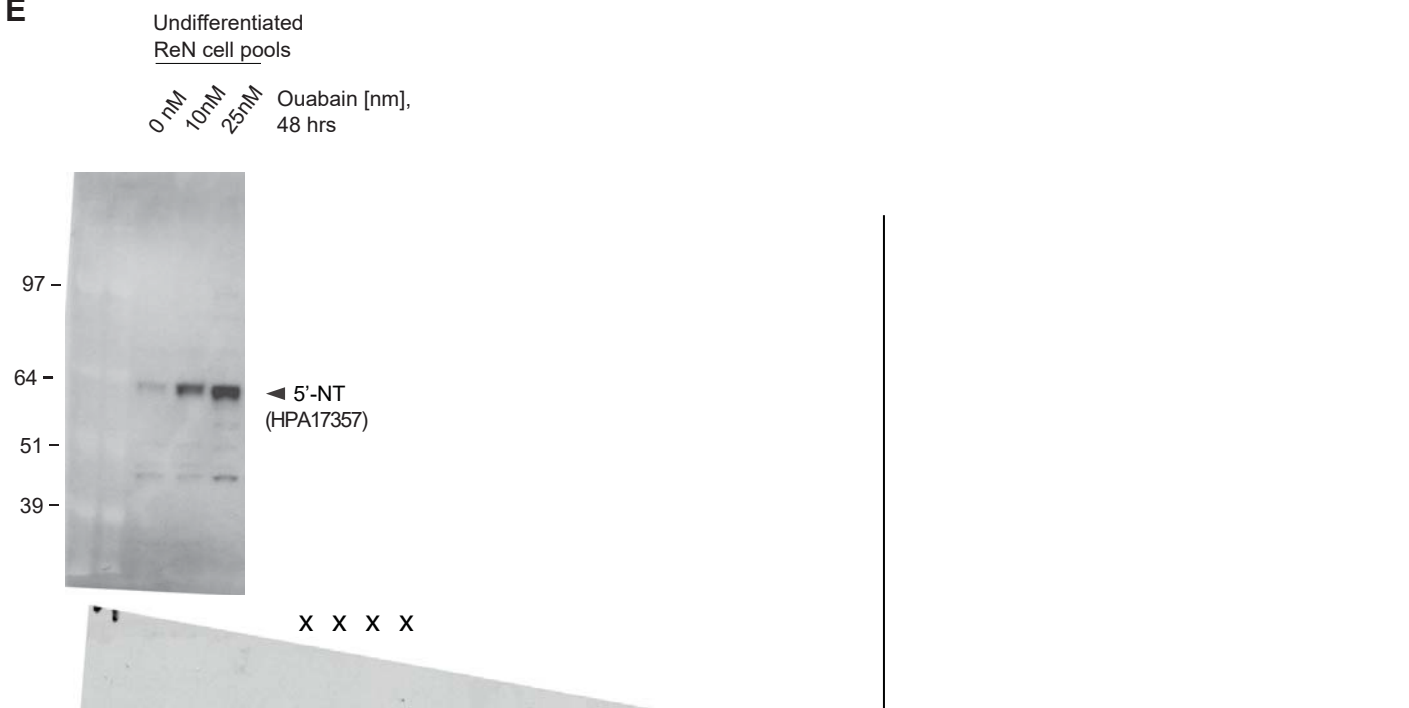

Supplement: S1 Raw images — (PDF) [file pone.0258682.s004.pdf]
