# Supplementary material for: The cellular prion protein interacts with and promotes the activity of Na,K-ATPases
Source: PLoS One. 2021 Nov 30;16(11):e0258682. doi: 10.1371/journal.pone.0258682 (PMC8631662; doi:10.1371/journal.pone.0258682)
Supplement: S1 Table — Levels of the tubulin alpha 1 subunit (Tuba1a), a protein we considered a non-specific interactor, were used to normalize enrichment levels following the assumption that differences in bulk surface in wild-type affinity matrices would skew relative binding of non-specific matrix interactors. Proteins preceding Tuba1a in this table exhibited PrP co-enrichment, with highest ranked proteins representing strong PrP candidate interactors. (PDF) [file pone.0258682.s003.pdf]

S1 Table. PrP interactors in mouse brain (full list; sorted on the basis of relative co-enrichment; including nonspecific interactors)

Accession	Description (curated)	Symbol	Coverage	Unique	Total Peptides	Total PSMs	# PSM wt1	# PSM wt2	# PSM wt3	# PSM ko	Score wt1	Score wt2	Score wt3	Score ko	# AAs	MW [kDa]	Calc. pI	Avg #PSM wt1-3	Avg #PSM wt1-3 minus #PSM ko
P04925	Major prion protein	Prnp	79.53%	13	18	525	135	197	174	16	175.5	240.5	201.9	1.8	254	28.0	9.33	168.7	152.3
A0A0A6YY91	Neural cell adhesion molecule 1 (Fragment)	Ncam1	52.07%	12	46	414	97	139	170	7	173.9	253.0	277.6	0.0	1089	116.3	4.83	135.3	128.5
E9QB01	Neural cell adhesion molecule 1	Ncam1	49.35%	4	37	305	70	108	123	3	118.6	198.8	222.5	0.0	849	93.6	4.93	100.3	96.9
Q6PIC6	Sodium/potassium-transporting ATPase subunit alpha-3	Atp1a3	56.76%	31	70	919	203	259	271	160	351.4	441.5	472.8	263.8	1013	111.6	5.41	244.3	84.6
P12960	Contactin-1	Cntn1	48.04%	23	43	248	42	78	110	15	52.5	102.0	147.5	4.0	1020	113.3	6.16	76.7	61.2
Q8VDN2	Sodium/potassium-transporting ATPase subunit alpha-1	Atp1a1	49.85%	19	57	561	142	155	146	101	235.3	254.8	267.6	165.7	1023	112.9	5.45	147.7	46.3
O35136	Neural cell adhesion molecule 2	Ncam2	57.59%	15	32	138	28	51	53	5	38.1	86.9	87.2	0.0	837	93.1	6.07	44.0	38.8
P05063	Fructose-bisphosphate aldolase C	Aldoc	55.65%	8	19	166	35	53	61	15	59.5	107.8	128.2	40.3	363	39.4	7.12	49.7	35.1
P60879	Synaptosomal-associated protein 25	Snap25	73.79%	12	20	131	41	39	42	8	75.1	63.5	70.8	0.0	206	23.3	4.77	40.7	32.9
A2AFG7	Neural cell adhesion molecule L1	L1cam	31.84%	11	27	131	34	39	49	8	49.9	56.8	98.8	1.7	1250	139.7	5.85	40.7	32.9
A6ZI44	Fructose-bisphosphate aldolase	Aldoa	71.05%	15	22	275	65	73	86	44	83.7	122.0	153.4	82.2	418	45.1	7.91	74.7	30.9
A0A087WR56	Neurofascin (Fragment)	Nfasc	47.97%	11	39	121	27	41	45	7	37.3	50.7	69.9	0.0	1159	130.4	6.95	37.7	30.8
Q02053	Ubiquitin-like modifier-activating enzyme 1	Uba1	49.62%	9	31	112	35	37	33	6	55.8	54.3	44.4	6.4	1058	117.7	5.66	35.0	29.0
Q6PIE5	Sodium/potassium-transporting ATPase subunit alpha-2	Atp1a2	62.84%	24	71	594	126	160	166	122	196.1	236.1	296.9	191.0	1020	112.1	5.55	150.7	28.7
P12023	Amyloid beta A4 protein	App	40.78%	13	28	127	32	35	47	11	29.5	54.9	56.1	0.0	770	86.7	4.79	38.0	26.8
K3W4T3	V-type proton ATPase subunit a	Atp6v0a1	45.47%	9	29	116	38	32	36	9	49.6	49.9	39.6	1.6	838	96.4	6.84	35.3	26.7
E9QN98	Inactive dipeptidyl peptidase 10	Dpp10	31.63%	9	19	78	26	28	24	0	41.6	53.2	42.1		800	91.1	6.48	26.0	26.0
Q99N28	Cell adhesion molecule 3	Cadm3	52.27%	6	14	77	24	22	31	0	35.1	29.9	46.8		396	42.9	5.8	25.7	25.7
P46096	Synaptotagmin-1	Syt1	50.36%	8	25	123	35	42	33	11	53.4	86.2	67.7	8.0	421	47.4	8.53	36.7	25.5
P14094	Sodium/potassium-transporting ATPase subunit beta-1	Atp1b1	43.09%	10	14	221	61	65	54	35	89.4	99.7	78.6	39.0	304	35.2	8.65	60.0	24.8
P20917-2	Myelin-associated glycoprotein	Mag	20.27%	1	11	71	20	27	24	0	33.1	46.6	55.4		582	64.2	5.08	23.7	23.7
Q810U4	Neuronal cell adhesion molecule	Nrcam	34.95%	8	33	122	18	34	55	13	11.5	18.5	43.9	0.0	1256	138.4	5.91	35.7	22.8
G3UYZ1	Immunoglobulin superfamily member 8	Igsf8	34.85%	8	11	68	13	22	33	0	33.3	64.3	66.8		548	58.1	7.91	22.7	22.7
P11499	Heat shock protein HSP 90-beta	Hsp90ab1	35.36%	5	20	120	34	35	36	13	63.1	52.6	63.9	15.1	724	83.2	5.03	35.0	22.1
G5E829	Plasma membrane calcium-transporting ATPase 1	Atp2b1	35.74%	8	41	227	46	57	79	39	92.3	97.1	130.9	53.3	1220	134.7	5.91	60.7	22.0
Q3ZB60	Mag protein (Fragment)	Mag	23.99%	1	12	66	18	26	22	0	24.1	42.9	50.5		567	62.5	5.39	22.0	22.0
Q0VF55	Calcium-transporting ATPase	Atp2b3	35.66%	5	41	186	49	45	58	29	71.0	58.9	60.3	34.9	1220	134.3	5.69	50.7	21.5
P61264	Syntaxin-1B	Stx1b	72.92%	8	21	107	26	34	35	10	56.6	66.3	76.2	1.8	288	33.2	5.38	31.7	21.4
Q80VM5	Dipeptidyl aminopeptidase-like protein 6	Dpp6	37.86%	10	27	84	21	25	32	5	28.4	38.4	30.2	0.0	803	91.2	6.48	26.0	20.8
Q8BG39	Synaptic vesicle glycoprotein 2B	Sv2b	34.26%	9	23	144	37	39	45	20	58.3	58.1	72.0	23.4	683	77.4	5.57	40.3	20.6
P43006	Excitatory amino acid transporter 2	Slc1a2	46.85%	10	23	232	57	68	58	42	75.6	92.3	66.6	77.2	572	62.0	6.68	61.0	18.9
P07901	Heat shock protein HSP 90-alpha	Hsp90aa1	32.47%	4	21	106	36	23	33	12	64.9	39.5	68.8	13.1	733	84.7	5.01	30.7	18.6
P17742	Peptidyl-prolyl cis-trans isomerase A	Ppia	66.46%	8	13	123	31	31	42	16	54.6	49.6	71.6	35.2	164	18.0	7.9	34.7	18.3
F7BJK1	Protein Pcdh1 (Fragment)	Pcdh1	28.51%	8	23	55	12	24	19	0	11.3	28.2	28.6		1077	116.4	5.08	18.3	18.3
Q9QYS2	Metabotropic glutamate receptor 3	Grm3	36.86%	6	26	83	21	29	25	7	10.8	19.4	17.8	0.0	879	99.0	7.75	25.0	18.1
F8WHB1	Calcium-transporting ATPase	Atp2b2	46.34%	13	50	247	53	68	72	46	96.5	95.5	83.8	46.0	1243	136.7	5.97	64.3	17.9
P17426-2	AP-2 complex subunit alpha-1	Ap2a1	30.79%	6	28	92	27	19	35	9	41.0	23.2	60.5	5.5	955	105.4	7.66	27.0	17.6
Q8K406	Leucine-rich repeat LGI family member 3	Lgi3	30.47%	11	18	99	21	32	33	11	52.9	62.6	66.3	17.6	548	61.8	8.16	28.7	17.5
Q9EQH3	Vacuolar protein sorting-associated protein 35	Vps35	43.97%	5	31	66	25	22	15	3	36.3	22.2	15.8	0.0	796	91.7	5.44	20.7	17.2
P14231	Sodium/potassium-transporting ATPase subunit beta-2	Atp1b2	68.62%	7	17	137	40	34	39	21	52.9	50.1	55.9	15.3	290	33.3	8.31	37.7	17.1
P62874	Guanine nucleotide-binding protein G(I)/G(S)/G(T) subunit beta-1	GNGNb1	48.53%	3	10	114	26	33	37	15	35.1	55.9	69.4	28.2	340	37.4	6	32.0	16.5
O54991	Contactin-associated protein 1	Cntnap1	21.01%	9	21	74	19	18	30	6	21.7	26.6	44.1	0.0	1385	156.2	7.06	22.3	16.3
P63318	Protein kinase C gamma type	Prkcg	33.43%	6	18	77	23	17	29	7	22.7	20.1	43.2	3.8	697	78.3	7.46	23.0	16.1
Q63810	Calcineurin subunit B type 1	Ppp3r1	62.94%	4	9	48	13	16	19	0	21.1	22.2	28.2		170	19.3	4.81	16.0	16.0
P08226	Apolipoprotein E	Apoe	54.98%	4	18	61	19	23	15	3	24.8	38.5	28.3	0.0	311	35.8	5.68	19.0	15.6
O35874	Neutral amino acid transporter A	Slc1a4	23.31%	1	7	46	19	15	12	0	19.9	26.5	20.8		532	56.0	5.87	15.3	15.3
P46097	Synaptotagmin-2	Syt2	57.82%	3	28	103	30	29	28	14	40.2	53.7	55.5	0.0	422	47.2	7.99	29.0	15.3
Q91V14-2	Solute carrier family 12 member 5	Slc12a5	32.74%	6	27	74	27	19	20	7	20.1	16.6	22.0	0.0	1115	123.5	6.24	22.0	15.1
P21836	Acetylcholinesterase	Ache	34.20%	7	16	56	17	16	20	3	12.8	29.3	40.2	2.2	614	68.1	6.33	17.7	15.1
E9Q1G8	Septin-7	43350	60.87%	8	27	106	32	36	21	15	43.1	32.6	23.0	7.5	437	50.6	8.57	29.7	15.1
Q9CPW0	Contactin-associated protein-like 2	Cntnap2	39.49%	9	37	91	24	23	31	11	25.7	31.9	35.0	0.0	1332	148.1	6.77	26.0	14.8
Q04447	Creatine kinase B-type	Ckb	65.09%	13	19	232	30	76	73	46	63.6	111.9	110.4	99.7	381	42.7	5.67	59.7	14.1
P20060	Beta-hexosaminidase subunit beta	Hexb	17.35%	6	9	42	17	11	14	0	15.1	14.5	14.6		536	61.1	8.12	14.0	14.0
P09411	Phosphoglycerate kinase 1	Pgk1	70.98%	15	30	213	53	54	58	41	78.1	91.8	99.5	60.5	417	44.5	7.9	55.0	13.8
P62746	Rho-related GTP-binding protein RhoB	Rhob	39.80%	5	8	41	13	16	12	0	7.6	16.4	3.8		196	22.1	5.24	13.7	13.7
Q9CWZ7	Gamma-soluble NSF attachment protein	Napg	49.04%	4	15	60	15	21	18	5	13.6	23.3	23.4	1.7	312	34.7	5.41	18.0	12.8
Q6GT24	Peroxiredoxin 6	Prdx6	58.04%	5	10	56	15	18	18	4	28.4	42.6	37.7	5.1	224	24.8	6.37	17.0	12.7
Q8C0M9	Isoaspartyl peptidase/L-asparaginase	Asrgl1	43.25%	5	9	38	15	7	16	0	36.0	10.3	35.6		326	33.9	7.65	12.7	12.7
Q9EPL2-2	Calsyntenin-1	Clstn1	30.44%	7	23	70	17	21	23	8	17.8	27.6	25.6	2.0	969	107.7	4.93	20.3	12.6
P01831	Thy-1 membrane glycoprotein	Thy1	56.79%	4	8	37	11	13	13	0	24.6	21.1	8.2		162	18.1	8.97	12.3	12.3
P35802	Neuronal membrane glycoprotein M6-a	Gpm6a	17.63%	2	6	37	9	15	13	0	17.7	12.0	14.5		278	31.1	5.27	12.3	12.3
O54983	Ketimine reductase mu-crystallin	Crym	28.12%	5	7	36	10	10	16	0	22.0	21.1	24.8		313	33.5	5.67	12.0	12.0
P28663	Beta-soluble NSF attachment protein	Napb	56.38%	6	19	75	23	21	20	9	26.9	25.3	39.2	0.0	298	33.5	5.47	21.3	11.9
Q6ZWQ0	Nesprin-2	Syne2	31.20%	2	153	275	63	84	61	58	13.3	20.1	14.0	25.2	6874	782.2	5.33	69.3	11.8
Q03517	Secretogranin-2	Scg2	42.30%	3	20	60	13	21	19	6	5.3	22.0	15.1	1.7	617	70.6	4.75	17.7	11.7
P68404-2	Protein kinase C beta type	Prkcb	44.58%	2	24	63	12	15	28	7	25.8	21.7	33.8	2.3	673	76.8	7.02	18.3	11.5
P09103	Protein disulfide-isomerase	P4hb	26.13%	7	12	34	6	11	17	0	6.6	11.8	20.7		509	57.0	4.88	11.3	11.3
Q6Q477	Calcium-transporting ATPase	Atp2b4	32.37%	3	32	123	24	40	34	21	46.7	38.0	55.4	12.3	1205	133.0	6.13	32.7	11.2
Q9R1V6-17	Disintegrin and metalloproteinase domain-containing protein 22	Adam22	35.54%	5	24	55	9	17	23	5	11.0	21.9	14.1	0.0	878	97.0	7.72	16.3	11.2
Q76MZ3	Serine/threonine-protein phosphatase 2A 65 kDa regulatory subunit A alpha isoform	Ppp2r1a	26.83%	8	15	44	14	11	16	3	12.5	10.7	16.2	1.9	589	65.3	5.11	13.7	11.1
O55042-2	Alpha-synuclein	Snca	80.99%	3	7	33	9	15	9	0	15.3	27.6	12.5		121	12.3	9.2	11.0	11.0
Q8K183	Pyridoxal kinase	Pdxk	63.78%	4	18	58	7	24	20	6	5.3	22.8	17.9	3.7	312	35.0	6.29	17.0	11.0
Q63844	Mitogen-activated protein kinase 3	Mapk3	37.11%	3	14	54	13	15	20	5	22.0	30.1	34.9	3.5	380	43.0	6.61	16.0	10.8
H7BWZ3	Actin-related protein 2/3 complex subunit 3	Arpc3	48.24%	3	5	32		15	17	0		22.9	24.8		170	19.6	8.6	10.7	10.7
P70202	Latexin	Lxn	24.77%	2	4	32	7	15	10	0	10.8	18.0	10.2		222	25.5	5.74	10.7	10.7
P62259	14-3-3 protein epsilon	Ywhae	62.35%	8	17	78	18	17	30	11	20.6	26.3	38.5	15.5	255	29.2	4.74	21.7	10.5
Q4VA93	Protein kinase C	Prkca	43.30%	2	24	70	17	17	25	9	25.5	18.3	36.2	0.0	672	76.8	7.05	19.7	10.2
F6RPJ9	Insulin-degrading enzyme (Fragment)	Ide	33.54%	9	22	66	21	17	18	9	25.9	19.3	18.2	0.0	987	114.2	6.27	18.7	10.1
Q80SW1	Putative adenosylhomocysteinase 2	Ahcyl1	47.74%	4	21	73	17	17	27	10	17.4	18.7	19.6	3.6	530	58.9	6.89	20.3	10.0
Q8C522	Endonuclease domain-containing 1 protein	Endod1	28.54%	3	10	30	8	10	12	0	8.4	13.7	7.1		501	55.2	6.16	10.0	10.0
P63085	Mitogen-activated protein kinase 1	Mapk1	50.00%	2	15	62	16	20	17	8	32.9	42.1	21.5	10.4	358	41.2	6.98	17.7	9.9
Q9QYG0-2	Protein NDRG2	Ndrg2	43.98%	5	13	47	8	18	16	4	15.7	23.4	23.8	0.0	357	39.3	5.63	14.0	9.7
Q9CQV8-2	14-3-3 protein beta/alpha	Ywhab	59.84%	4	15	104	32	21	30	18	43.5	40.2	52.4	29.1	244	27.8	4.83	27.7	9.6
Q923J1	Transient receptor potential cation channel subfamily M member 7	Trpm7	33.92%	1	40	61	15	22	15	8	0.0	2.2	0.0	0.0	1863	212.3	7.8	17.3	9.6
Q61735-2	Leukocyte surface antigen CD47	Cd47	46.60%	2	12	28	11	10	7	0	15.4	20.7	11.9		324	35.3	8.25	9.3	9.3
Q8R5M8-4	Cell adhesion molecule 1	Cadm1	34.77%	3	11	28	5	12	11	0	5.9	11.0	12.9		417	45.8	5.12	9.3	9.3
G3UXX3	Sepiapterin reductase	Spr	30.14%	3	5	28	11	9	8	0	16.6	17.9	11.0		219	23.3	6.21	9.3	9.3
P61161	Actin-related protein 2	Actr2	49.24%	10	20	171	46	44	41	34	62.6	66.8	74.1	43.2	394	44.7	6.74	43.7	9.3
P09528	Ferritin heavy chain	Fth1	89.56%	3	10	42	10	13	15	3	10.5	15.7	11.0	0.0	182	21.1	5.88	12.7	9.2
A3KGE4	Noelin	Olfm1	19.68%	1	11	27	7	8	12	0	4.3	5.5	5.9		442	51.0	6.54	9.0	9.0
Q5ICG5	Long chain acyl-CoA synthetase 6 isoform 3	Acsl6	38.59%	6	19	55	15	14	18	7	21.9	15.5	26.8	7.1	697	77.9	7.24	15.7	8.8
P17183	Gamma-enolase	Eno2	44.24%	7	14	105	21	29	33	19	43.8	67.7	62.5	37.3	434	47.3	5.11	27.7	8.8
O88643	Serine/threonine-protein kinase PAK 1	Pak1	40.55%	6	15	44	15	11	13	4	14.3	14.3	16.2	2.0	545	60.7	5.74	13.0	8.7
P59279	Ras-related protein Rab-2B	Rab2b	35.65%	1	5	28	5	11	10	0	2.8	25.5	25.0		216	24.2	6.68	8.7	8.7
P18572	Basigin	Bsg	29.31%	3	7	26	11	10	5	0	2.0	9.5	4.2		389	42.4	5.85	8.7	8.7
Q6P5F6	Zinc transporter ZIP10	Slc39a10	23.29%	2	15	40	10	15	11	3	7.6	15.5	15.4	0.0	833	94.3	6.71	12.0	8.6
P63213	Guanine nucleotide-binding protein G(I)/G(S)/G(O) subunit gamma-2	GNGNg2	69.01%	3	5	25	8	8	9	0	17.3	14.5	14.9		71	7.8	7.99	8.3	8.3
F7C0H6	CB1 cannabinoid receptor-interacting protein 1 (Fragment)	Cnrip1	53.26%	2	7	25	5	10	10	0	8.5	12.4	9.9		184	20.7	9.36	8.3	8.3
P61021	Ras-related protein Rab-5B	Rab5b	47.91%	2	8	25	9	8	8	0	11.6	5.9	5.7		215	23.7	8.13	8.3	8.3
Q9Z140	Copine-6	Cpne6	20.83%	4	10	25	8	8	9	0	10.7	13.8	12.9		557	61.7	5.59	8.3	8.3
Q99PT1	Rho GDP-dissociation inhibitor 1	Arhgdia	19.12%	3	4	25	9	7	9	0	16.7	14.6	18.5		204	23.4	5.2	8.3	8.3
P63011	Ras-related protein Rab-3A	Rab3a	77.27%	3	13	82	26	21	19	14	36.7	34.4	18.9	12.4	220	25.0	5.03	22.0	8.3
F6YY69	14-3-3 protein theta (Fragment)	Ywhaq	40.70%	4	13	78	23	20	20	13	44.6	42.2	40.6	21.7	285	32.3	5.94	21.0	8.1
Q8BLQ9-3	Cell adhesion molecule 2	Cadm2	32.91%	4	9	24		10	14	0		19.3	21.9		395	43.5	5.33	8.0	8.0
A2AQR0	Glycerol-3-phosphate dehydrogenase	Gpd2	37.45%	6	19	45	8	13	18	5	7.6	3.8	10.8	1.7	745	82.8	6.42	13.0	7.8
A2ALV3	Endophilin-A1	Sh3gl2	51.74%	5	16	59	12	20	17	9	16.7	22.6	25.3	7.8	431	48.3	5.69	16.3	7.7
O35526	Syntaxin-1A	Stx1a	33.68%	4	10	41	12	8	16	4	15.3	11.6	34.6	2.6	288	33.0	5.24	12.0	7.7
P68510	14-3-3 protein eta	Ywhah	49.59%	3	19	91	24	20	28	16	27.5	32.1	38.1	12.2	246	28.2	4.89	24.0	7.7
Q91V12	Cytosolic acyl coenzyme A thioester hydrolase	Acot7	60.89%	3	14	62	12	17	22	9	21.7	27.0	35.7	19.4	381	42.5	8.68	17.0	7.6
J3QNE4	Protein E230008N13Rik	E230008N13Rik	39.18%	1	46	94	19	25	30	17	4.1	0.0	5.1	2.4	1664	195.7	5.31	24.7	7.5
P59242	Cingulin	GNGN	44.42%	1	40	76	18	16	27	13	5.1	3.9	10.4	2.1	1191	136.4	5.91	20.3	7.4
Q5SVG5	AP-1 complex subunit beta-1	Ap1b1	25.55%	1	22	58	17	10	21	9	25.4	15.4	33.1	5.8	916	101.1	5.22	16.0	7.4
P19157	Glutathione S-transferase P 1	Gstp1	51.43%	1	9	40	8	16	11	4	0.0	5.2	0.0	0.0	210	23.6	7.87	11.7	7.4
O55143	Sarcoplasmic/endoplasmic reticulum calcium ATPase 2	Atp2a2	31.80%	10	30	133	33	36	33	27	50.4	61.3	45.9	46.2	1044	114.8	5.34	34.0	7.4
Q9CQD1	Ras-related protein Rab-5A	Rab5a	63.26%	2	10	22	7	8	7	0	8.2	7.7	9.1		215	23.6	8.15	7.3	7.3
Q91X78	Erlin-1	Erlin1	49.71%	1	12	22	8	8	6	0	1.9	0.0	0.0		346	38.9	7.21	7.3	7.3
O35129	Prohibitin-2	Phb2	35.45%	3	9	22	7	4	11	0	9.1	0.0	19.7		299	33.3	9.83	7.3	7.3
A0A087WSB2	Leucine-rich repeat-containing protein 28 (Fragment)	Lrrc28	33.96%	1	4	22		16	6	0		6.6	1.7		159	18.3	8.28	7.3	7.3
P31648	Sodium- and chloride-dependent GABA transporter 1	Slc6a1	13.36%	1	4	22		12	10	0		17.9	11.1		599	67.0	7.96	7.3	7.3
Q8K0U4	Heat shock 70 kDa protein 12A	Hspa12a	44.44%	10	22	72	15	21	22	12	28.1	45.5	37.4	15.8	675	74.8	6.77	19.3	7.3
Q9EQF6	Dihydropyrimidinase-related protein 5	Dpysl5	38.83%	7	19	72	16	21	21	12	20.8	18.2	23.7	13.4	564	61.5	7.09	19.3	7.3
Q6P5U7	NACHT and WD repeat domain-containing protein 2	Nwd2	23.71%	1	21	36	15	10	7	3	0.0	0.0	0.0	0.0	1742	197.3	6.15	10.7	7.2
P17427	AP-2 complex subunit alpha-2	Ap2a2	34.65%	3	24	61	20	15	15	9	20.9	10.5	34.0	0.0	938	104.0	6.93	16.7	7.2
Q8BGT8	Phytanoyl-CoA hydroxylase-interacting protein-like	Phyhipl	49.33%	3	13	43	12	11	14	5	11.8	7.8	10.5	1.7	375	42.3	6.35	12.3	7.2
Q6PHU5-2	Sortilin	Sort1	34.85%	3	20	43	13	8	16	5	17.9	11.2	13.1	0.0	858	94.8	5.99	12.3	7.2
B0V2N1-6	Receptor-type tyrosine-protein phosphatase S	Ptprs	24.56%	2	24	50	15	10	17	7	21.5	0.0	0.0	1.7	1861	206.7	7.28	14.0	7.1
P00920	Carbonic anhydrase 2	Ca2	53.46%	4	8	21		8	13	0		4.3	14.1		260	29.0	7.01	7.0	7.0
Q71M36	Chondroitin sulfate proteoglycan 5	Cspg5	23.50%	3	7	21		12	9	0		16.4	13.6		566	60.4	4.54	7.0	7.0
P06151	L-lactate dehydrogenase A chain	Ldha	46.39%	5	16	85	22	25	20	15	26.7	35.9	24.9	19.5	332	36.5	7.74	22.3	6.9
P10649	Glutathione S-transferase Mu 1	Gstm1	68.81%	6	17	110	30	31	24	21	59.2	57.1	51.8	22.8	218	26.0	7.94	28.3	6.9
O88307	Sortilin-related receptor	Sorl1	35.67%	4	55	110	25	26	34	21	10.5	3.8	5.8	0.0	2215	246.9	5.54	28.3	6.9
Q9R111	Guanine deaminase	Gda	35.68%	7	13	42	10	15	11	5	23.9	26.8	18.7	9.5	454	51.0	5.53	12.0	6.8
O70318	Band 4.1-like protein 2	Epb41l2	34.82%	1	29	56	12	13	21	9	4.4	5.2	5.9	8.1	988	109.9	5.43	15.3	6.7
Q8BLE7	Vesicular glutamate transporter 2	Slc17a6	21.65%	2	9	23	9	5	6	0	4.5	0.0	2.5		582	64.5	6.68	6.7	6.7
Q8R422	CD109 antigen	Cd109	19.90%	1	16	20	4	9	7	0	1.9	1.6	3.5		1442	161.6	5.57	6.7	6.7
P63001	Ras-related C3 botulinum toxin substrate 1	Rac1	45.31%	5	9	59	16	12	20	9	34.0	23.6	37.0	5.9	192	21.4	8.5	16.0	6.6
Q11011	Puromycin-sensitive aminopeptidase	Npepps	36.20%	12	28	98	27	24	25	19	48.9	41.6	45.8	25.2	920	103.3	5.9	25.3	6.4
G3UX26	Voltage-dependent anion-selective channel protein 2 (Fragment)	Vdac2	35.34%	3	6	19		6	13	0		6.0	10.4		283	30.4	7.58	6.3	6.3
Q9CPU0	Lactoylglutathione lyase	Glo1	28.80%	3	6	19		9	10	0		15.0	14.5		184	20.8	5.47	6.3	6.3
E9Q912	Protein Rap1gds1	Rap1gds1	28.67%	3	17	44	11	14	12	6	8.4	9.5	15.3	0.0	607	66.0	5.35	12.3	6.3
Q3UHF7	Transcription factor HIVEP2	Hivep2	30.25%	2	51	101	25	27	26	20	1.7	5.2	1.7	1.6	2430	266.5	6.96	26.0	6.2
P50396	Rab GDP dissociation inhibitor alpha	Gdi1	50.34%	7	21	58	15	16	16	9	13.4	16.7	23.6	4.0	447	50.5	5.08	15.7	6.2
G5E8H0	Villin-like protein	Vill	29.03%	1	16	29	9	9	8	3	5.7	3.6	5.9	0.0	775	86.9	6.27	8.7	6.1
E9PZM8	Neuronal pentraxin receptor	Nptxr	55.38%	3	12	36	9	9	13	4	10.4	15.4	23.3	0.0	493	52.3	6.13	10.3	6.0
Q91ZZ3	Beta-synuclein	Sncb	46.62%	2	6	21	7	11		0	15.4	20.0			133	14.0	4.37	6.0	6.0
Q9CQR4	Acyl-coenzyme A thioesterase 13	Acot13	42.86%	1	7	18	6	9	3	0	8.3	7.6	1.7		140	15.2	8.82	6.0	6.0
P63044	Vesicle-associated membrane protein 2	Vamp2	38.79%	3	4	21	11		7	0	25.9		17.6		116	12.7	8.13	6.0	6.0
P70296	Phosphatidylethanolamine-binding protein 1	Pebp1	37.97%	4	4	18	6		12	0	13.0		19.8		187	20.8	5.4	6.0	6.0
Q9CR16	Peptidyl-prolyl cis-trans isomerase D	Ppid	32.43%	1	9	18	6	7	5	0	1.7	5.5	3.6		370	40.7	7.43	6.0	6.0
D3YVN7	Elongation factor Tu	Gm9755	57.96%	13	23	118	33	23	34	24	51.4	28.6	61.9	46.5	452	49.5	7.56	30.0	5.9
P10852-2	4F2 cell-surface antigen heavy chain	Slc3a2	41.95%	7	20	75	17	17	25	14	19.4	24.7	36.9	12.2	565	62.2	5.5	19.7	5.9
P53986	Monocarboxylate transporter 1	Slc16a1	31.64%	1	9	32		12	16	3		2.5	3.0	3.4	493	53.2	7.47	9.3	5.9
P21278	Guanine nucleotide-binding protein subunit alpha-11	GNGNGNA11_MO	41.23%	3	11	39	12	9	12	5	20.9	8.4	21.5	6.3	359	42.0	5.97	11.0	5.8
Q8C419	Probable G-protein coupled receptor 158	Gpr158	34.92%	2	30	60	14	16	18	10	11.5	8.6	20.7	5.2	1200	134.3	8.09	16.0	5.7
Q9D1E6	Tubulin-folding cofactor B	Tbcb	34.43%	1	7	17	4	6	7	0	3.5	1.7	6.0		244	27.4	5.24	5.7	5.7
Q8BG33	Neurotrimin	Ntm	36.92%	5	9	31		10	17	3		13.5	22.0	2.1	344	37.9	7.85	9.0	5.6
P68368	Tubulin alpha-4A chain	Tuba4a	70.31%	8	28	596	133	152	149	139	210.5	296.2	270.6	241.7	448	49.9	5.06	144.7	5.5
O89053	Coronin-1A	Coro1a	23.21%	2	10	45	14	11	12	7	14.7	13.1	13.9	4.4	461	51.0	6.48	12.3	5.5
Q8BV79	TPR and ankyrin repeat-containing protein 1	Trank1	29.18%	1	60	102	25	35	18	21	0.0	7.3	1.7	0.0	2999	343.1	7.81	26.0	5.4
Q8BKN5	Gamma-tubulin complex component 5	Tubgcp5	28.13%	1	20	34	4	15	10	4	1.8	6.1	7.5	0.0	1024	117.9	5.99	9.7	5.4
Q60625	Intercellular adhesion molecule 5	Icam5	32.28%	7	18	59	14	15	18	10	9.5	12.4	31.5	7.8	917	96.9	6.32	15.7	5.4
Q91VU0	Protein FAM3C	Fam3c	53.74%	1	9	18	7	3	6	0	1.6	0.0	3.7		227	24.7	8.29	5.3	5.3
Q3TYE5	Limbic system-associated membrane protein	Lsamp	32.25%	1	8	16	7	9		0	6.7	4.4			338	37.3	6.68	5.3	5.3
Q9CY27	Very-long-chain enoyl-CoA reductase	Tecr	28.57%	3	5	16	9		7	0	8.4		11.3		308	36.1	9.55	5.3	5.3
P58252	Elongation factor 2	Eef2	33.80%	5	17	66	19	13	20	12	35.5	17.5	29.2	10.6	858	95.3	6.83	17.3	5.3
D3YWF6	Ubiquitin thioesterase OTUB1	Otub1	37.34%	4	8	55	13	21	10	9	25.6	38.1	18.8	20.6	241	28.0	5.29	14.7	5.2
P21279	Guanine nucleotide-binding protein G(q) subunit alpha	GNGNGNAQ_MO	37.60%	2	9	37	11	13	7	5	25.1	16.4	14.9	6.3	359	42.1	5.68	10.3	5.2
Q7TSJ2	Microtubule-associated protein 6	Map6	59.38%	4	35	69	16	20	18	13	11.2	23.3	12.8	0.0	906	96.4	9.5	18.0	5.1
Q69ZK9	Neuroligin-2	GNGNGN2_MOU	13.16%	3	8	26	6	9	8	3	0.0	10.8	7.7	0.0	836	90.9	6.18	7.7	5.1
Q9CZT8	Ras-related protein Rab-3B	Rab3b	78.54%	2	13	51	17	11	13	9	21.3	19.1	10.7	17.8	219	24.7	5.11	13.7	5.1
P80316	T-complex protein 1 subunit epsilon	Cct5	49.72%	4	21	51	18	7	16	9	16.8	1.9	14.3	14.4	541	59.6	6.02	13.7	5.1
O35098-2	Dihydropyrimidinase-related protein 4	Dpysl4	41.65%	4	20	76	14	21	24	15	15.9	28.3	44.3	8.1	593	64.3	7.09	19.7	5.1
E9Q1S3	Protein transport protein Sec23A	Sec23a	53.80%	1	25	58	14	23	9	10	7.5	6.9	11.4	1.8	736	82.9	7.46	15.3	5.0
A2A813	Protein deglycase DJ-1	Park7	48.57%	3	7	58	17	14	15	10	16.8	19.9	27.8	11.1	175	18.5	6.54	15.3	5.0
P47757-2	F-actin-capping protein subunit beta	Capzb	49.26%	1	10	17	6	6	3	0	4.0	1.8	0.0		272	30.6	6	5.0	5.0
D3YWS7	Fucose mutarotase	Fuom	30.60%	2	4	15		8	7	0		10.3	7.3		134	14.8	5.31	5.0	5.0
Q80TL4-2	Protein KIAA1045	Kiaa1045	21.76%	2	8	15		7	8	0		9.1	8.4		363	41.3	5.45	5.0	5.0
Q3U4W8	Ubiquitin carboxyl-terminal hydrolase	Usp5	31.02%	5	19	65	19	14	18	12	17.1	6.3	17.7	4.3	835	93.3	5.06	17.0	5.0
P61982	14-3-3 protein gamma	Ywhag	38.87%	4	14	90	19	21	29	18	41.5	43.5	54.1	26.9	247	28.3	4.89	23.0	5.0
Q9JIA1	Leucine-rich glioma-inactivated protein 1	Lgi1	33.21%	4	16	47	14	8	16	8	15.7	11.0	12.5	1.7	557	63.6	8.02	12.7	4.9
E9Q8N8	Anion exchange protein	Slc4a4	20.38%	1	18	36	8	13	9	5	1.6	1.8	3.4	0.0	1094	123.0	6.89	10.0	4.8
Q61885	Myelin-oligodendrocyte glycoprotein	Mog	61.79%	6	11	61	14	17	17	11	21.0	37.9	28.3	10.5	246	28.3	7.96	16.0	4.8
D3Z656	Synaptojanin-1	Synj1	33.73%	3	33	86	24	24	18	17	16.9	16.5	19.3	7.3	1607	176.2	7.72	22.0	4.8
Q5SRA0	Disintegrin and metalloproteinase domain-containing protein 23	Adam23	33.81%	2	21	43	8	10	17	7	10.4	10.2	14.1	0.0	837	93.0	7.58	11.7	4.8
P63328	Serine/threonine-protein phosphatase 2B catalytic subunit alpha isoform	Ppp3ca	32.25%	7	11	50	19	9	12	9	21.7	17.4	18.1	6.5	521	58.6	5.86	13.3	4.7
Q80V24	Transcription cofactor vestigial-like protein 4	Vgll4	43.90%	1	7	14	7		7	0	0.0		0.0		287	31.0	8.62	4.7	4.7
P47754	F-actin-capping protein subunit alpha-2	Capza2	34.97%	2	7	17	6	8		0	0.0	3.4			286	32.9	5.85	4.7	4.7
Q61206	Platelet-activating factor acetylhydrolase IB subunit beta	Pafah1b2	33.19%	2	8	14		8	6	0		8.6	6.1		229	25.6	5.92	4.7	4.7
P60766	Cell division control protein 42 homolog	Cdc42	32.46%	2	5	19	6		8	0	12.5		11.0		191	21.2	6.55	4.7	4.7
Q9JLI2	Collagen type V alpha 3 chain	Col5a3	32.66%	1	35	64	13	15	22	12	6.9	10.9	10.7	5.8	1739	171.9	6.8	16.7	4.6
P62761	Visinin-like protein 1	Vsnl1	53.40%	6	11	46	10	12	15	8	14.4	12.6	28.4	6.3	191	22.1	5.15	12.3	4.6
P47708	Rabphilin-3A	Rph3a	49.49%	1	26	78	18	17	25	15	10.7	13.3	10.4	0.0	681	75.4	8.27	20.0	4.5
B2RWS6	Histone acetyltransferase p300	Ep300	29.56%	1	46	78	9	25	26	15	2.1	4.6	0.0	0.0	2412	263.1	8.54	20.0	4.5
Q9D394-3	Protein RUFY3	Rufy3	49.90%	1	23	49	9	13	17	9	8.0	7.3	8.5	6.4	487	55.1	5.2	13.0	4.4
Q14BI2	Metabotropic glutamate receptor 2	Grm2	17.89%	4	15	31		9	17	4		10.3	20.6	0.0	872	95.8	8.06	8.7	4.4
P27773	Protein disulfide-isomerase A3	Pdia3	30.30%	1	11	13		6	7	0		4.0	2.1		505	56.6	6.21	4.3	4.3
Q9R1Q8	Transgelin-3	Tagln3	66.33%	5	16	63	14	24	11	12	20.2	24.0	16.5	1.9	199	22.5	7.33	16.3	4.3
Q3UHL1	CaM kinase-like vesicle-associated protein	Camkv	39.84%	6	17	45	13	9	14	8	13.3	6.0	19.5	7.8	512	54.8	5.54	12.0	4.3
Q8VDC1	FYVE and coiled-coil domain-containing protein 1	Fyco1	23.66%	1	26	45	7	12	17	8	7.4	4.2	9.5	3.5	1437	162.2	4.98	12.0	4.3
P58022	Lysyl oxidase homolog 2	Loxl2	23.58%	1	14	45	12	15	9	8	8.0	3.8	0.0	1.7	776	86.9	6.57	12.0	4.3
O70161-3	Phosphatidylinositol 4-phosphate 5-kinase type-1 gamma	Pip5k1c	23.46%	4	11	27	4	10	9	3	4.2	16.7	11.8	0.0	635	69.5	5.73	7.7	4.2
